# Modified tree-based selection in hierarchical mixed-effect models with trees: A simulation study and real-data application

**DOI:** 10.1016/j.mex.2025.103312

**Published:** 2025-04-12

**Authors:** Asrirawan, Khairil Anwar Notodiputro, Budi Susetyo, Sachnaz Desta Oktarina

**Affiliations:** a Department of Statistics, Faculty of Mathematics and Natural Sciences, Universitas Sulawesi Barat, Indonesia; b Department of Statistics and Data Sciences, Faculty of Mathematics and Natural Sciences, IPB University, Indonesia

**Keywords:** Regression tree, Evolutionary learning, Conditional inference, Hierarchical data, Machine learning, 3Trees-EvTree and 3Trees-CTree

## Abstract

Hierarchical mixed-effects models with three trees—3Trees models—are a new advanced statistical learning approach in mixed-effect modeling. These methods utilize the classification and regression trees (CART) algorithm to select the best tree through a backfitting algorithm. However, this algorithm relies on a greedy approach, making the trees prone to overfitting, biased in split selection, and often far from the optimal solution, ultimately affecting model performance. Two novel methods are proposed—3Trees-EvTree and 3Trees-CTree—to address these limitations. The proposed methods are compared with the available methods through several simulation exercises in different settings and real datasets. The simulation study confirms that the 3Trees-EvTree method performs well compared to the previous method in terms of parameter estimation and prediction accuracy under clusMSE and clusPMSE. Meanwhile, the 3Trees-CTree model performs well in low-correlation scenarios and the semilinear function. In addition, the proposed methods also reveal that the results of actual application confirm their superiority over other competing methods. Some highlights of the proposed method are:

• 3Trees-EvTree and 3Trees-CTree model to improve prediction accuracy and to reduce bias of 3Trees model are presented
• MSE, ClusMSE, PMSE, ClusPMSE, and bias criteria are used to evaluate model performance
• Applied to estimate and predict household expenditure per capita dataset

## Graphical abstract

**Figure:**
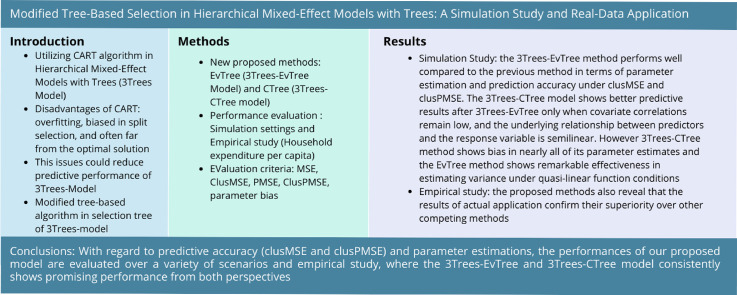

Specifications table

**Table:** 

Subject area:	Mathematics and Statistics
More specific subject area:	Statistical learning, machine learning, mixed-effects model
Name of your method:	3Trees-EvTree and 3Trees-CTree
Name and reference of original method:	A. Gottard, G. Vannucci, L. Grilli, C. Rampichini, Mixed-effect models with trees, Adv. Data Anal. Classif. 17 (2) (2023) 431–461, doi: 10.1007/s11634-022-00509–3.
Resource availability:	household expenditure per capita dataset of West Java and its predictor variables (Individual level and village level) distributed among 27 regencies and cities a can be accessed on the BPS official website (https://silastik.bps.go.id)

## Background

Linear mixed-effects models (LMMs), also known as multilevel or hierarchical or random effects models [1], have become increasingly popular among statisticians and data scientists. Due to its ability to analyze dependency data structures such as clustering/nested or longitudinal data, this model has been widely applied across various fields, such as education [[2], [3], [4]], biostatistics [5,6], psychology [7,8], and economics [[9], [10], [11]]. These models are designed to capture both the variance between units at the higher level (e.g., differences between schools, regions, or countries) and the intra-unit dependencies within those higher-level units (e.g., students within a school, patients within a healthcare facility, households within a region).

At present, the estimation procedures of hierarchical mixed-effect models have been extended to machine learning methods. These approaches are considered more powerful as they are not constrained by the assumptions typically faced in statistical models [12]. Hajjem et al. [13] proposed the Mixed-Effect Regression Tree model (MERT) designed to handle clustered or hierarchical data. This model extends the standard regression tree by replacing the linear fixed effects component of LMMs models with a tree structure, utilizing the CART algorithm. Then, a portion of the random effects is estimated based on a node-invariant linear structure. Sela and Simonoff [14] also discussed a data mining technique intended for clustered and longitudinal data with numerical response variables. This approach integrates the flexibility of data mining with the distinct characteristics of clustered or longitudinal datasets. Furthermore, their simulation results confirm that RE-EM Trees generally outperform classical linear mixed models. Additionally, the existing literature has been widely extended to numerous variants and extensions, allowing response or target outcome distributions, for instance, to deviate from the Gaussian assumption (see Fontana et al. [15] and Pellagatti et al. [16]).

Regression trees are highly effective in estimating the linear components of mixed-effects models and generating accurate predictions. However, they require more splits to build a sufficiently complex tree structure, such as the following semi-linear functions. This condition added complexity can substantially influence the model's predictive performance (see Vannucci [17] and Hastie et al. [18]). To overcome this issue, Gottard et al. [19] proposed a new method called hierarchical mixed-effect with trees models (known as the 3Trees model). This model integrates a linear part and a regression tree in a single equation. The 3Trees model also effectively balances both the predictive and generative perspectives. The linear term helps to maintain the three-tree structures as short as possible, while the tree components function as weak learners of prediction. The outcome model is simpler to understand than a single tree or a random forest. Additionally, it delivers better predictive results than a linear mixed-effects model. The 3Trees model employs three separate decision trees to capture non-linearity and interactions among explanatory variables effectively. All three trees are built upon the foundational CART algorithm [19]. The estimation of the 3Trees model parameters is performed through an iterative procedure, similar to backfitting, which alternates between fitting the linear component and the tree components.

Despite the CART algorithm has shown excellent performance in regression and classification settings [[20], [21], [22]], including applying to the 3Trees model [19], it has been identified in various studies for its notable limitations (See e.g. Grubinger et al. [23] and Hothorn et al. [24]). This approach has three fundamental problems: overfitting, a selection bias towards covariates [24], and local optimal solutions [23]. CART tends to overfit because it performs an exhaustive search over all possible splits, which maximizes an information measure of node impurity without considering other aspects. The selection bias in CART arises because it tends to favor covariates with many possible splits, primarily when the measurement scales differ between covariates. CART is biased towards selecting covariates with more potential split points, which can lead to skewed results when covariates are measured on different scales. The CART algorithm uses a forward stepwise search that selects the best split at each step to maximize the homogeneity of child nodes, but it does so without considering the overall tree structure, leading to only locally optimal solutions. Since CART focuses on optimizing splits one step at a time rather than globally, it often fails to find the best possible tree structure across all splits, resulting in suboptimal, locally optimal trees. The CART algorithm also uses a greedy approach, resulting in trees that are far from the optimal solution for some problems. Ultimately, this could potentially reduce the performance of the 3Trees model.

This paper addresses the issues by modifying the regression tree method for selecting the best tree in the 3Trees algorithm, utilizing alternative single-tree approaches. Two new 3Trees models called 3Trees-EvTree and 3Trees-CTree algorithms are proposed, which mainly use the EvTree and CTree methods, respectively, to fit tree components in the 3Trees model. The EvTree algorithm allows it to avoid the locally optimal solutions of CART's greedy heuristic approach. This results in potentially more compact and accurate models, especially for complex datasets. Meanwhile, the CTree algorithm avoids the expected bias in CART towards variables with many possible splits or missing values, and offers well-defined stopping criteria based on statistical hypothesis testing. Compared with the previous algorithm, the newly proposed methods can potentially enhance the model performance of the 3Trees. Both methods are expected to have smaller prediction errors compared to the previous approach, both in terms of errors during the tree selection phase and the prediction errors from the best model estimation stage in the 3Trees backfitting algorithm.

## Method details

### Hierarchical mixed-effects model with trees (3Trees)

The 3Trees model, a hierarchical mixed-effects model involving three trees, was first proposed by Gottard et al. [19]. This additive model combines linear components with three decision trees within a mixed-effects framework. The first tree aims to capture interactions and non-linearity at the individual level (level 1), the second tree captures interactions and non-linearity at the cluster/group level (level 2), and the third tree focuses on capturing cross-level interactions and non-linearity between levels 1 and 2. The linear component helps to keep the tree structures as concise as possible. In contrast, the three trees act as weak learners, allowing the model to address complex hierarchical data structures flexibly.

Suppose that the data are $\left(Y_{ij},X_{ij},Z_{j}\right),\;i=1,2,\ldots ,n_{j},\;j\;=1,2,\ldots ,J,\;n_{total}=\sum_{j=1}^{J}n_{j}$, where i is an index for individual and j is an index for cluster. $Y_{ij}$ denotes response variable. Hierarchical mixed-effects model with trees (Eq. (1)) is

(1) $$Y_{ij}=X_{ij}^{T}\beta +Z_{j}^{T}\gamma +\sum_{t=1}^{3}T_{t}\left(h_{tij}\right)+u_{j}+\varepsilon_{ij}$$

with $T_{t}\left(t=1,2,3\right)$ estimated using the CART algorithm. Note that Eq. (1), when employing the tree selection process using CART, is referred to as the 3Trees-CART model. Meanwhile, if the tree selection process utilizes EvTree or CTree, it is referred to as the 3Trees-EvTree and 3Trees-CTree models, respectively. β and γ represent the fixed-effect parameters for observation units and group units, respectively. The matrix X at the individual level is of size $n_{tot}\times p_{1}$ with $X_{ij}$ rows, while Z at the group level is of size $n_{tot}\times p_{2}$ with $Z_{j}$ rows. The within-groups errors $\varepsilon_{ij}$ and the random effects $u_{j}$ are assumed to be independent, identically distributed (iid) stochastic errors, each following the distributions $N(0,\sigma_{\varepsilon }^{2})$ and $N(0,\sigma_{u}^{2})$, and both errors are assumed to be independent of each other. The random component $b_{j}=\beta_{0}+u_{j}$ represents the random intercept for each group *j*, with $b_{j}⊥b_{j}^{T}$ for each $j\neq j^{T}\in \left\{1,2,\ldots ,\;J\right\}$.

Specifically, $h_{1ij}\subseteq X_{ij}$ is the partition space for level 1 predictors, $h_{2ij}\subseteq Z_{j}$ is the partition space for level 2 predictors, and $h_{3ij}\subseteq \left(X_{ij}\cup Z_{j}\right)$ is the combined partition space for both level 1 and level 2 predictors. The model can be expressed as an additive model equation (Eq. (2)):

(2) $$Y_{ij}=X_{ij}^{T}\beta +Z_{j}^{T}\gamma +\sum_{t=1}^{3}\sum_{m=1}^{M_{t}}\mu_{tm}I\left\{h_{tij}\epsilon R_{tm}\right\}+u_{j}+\varepsilon_{ij}$$

with $R_{tm}=\left[R_{t1},\;R_{t2},\ldots ,R_{tM_{t}}\right]$ representing the tree partition region, where each tree is a factor of the category $M_{t}$. This partition is identified by multiplying all dummy variables determined by binary splits along the path from the root node to each leaf in the tree. The tree parameters are denoted by $\mu_{tm}$, with $m=1,2,\ldots ,M_{t},\;t=1,2,3$ representing the tree structure. The linear component parameters include β and γ, while the variance parameters for the random effects and error components are $\sigma_{u}^{2}$ and $\sigma_{\varepsilon }^{2}$, respectively. The variance components for random effects are estimated using Restricted Maximum Likelihood (REML).

Gottard et al. [19] developed the 3Trees algorithm (Alg-3Trees) for estimating and predicting the 3Trees model. The pseudocode for the Alg-3Trees algorithm is presented in Algorithm 1. This algorithm consists of two major stages: the selection stage and the estimation stage. During the selection stage, the process identifies the best tree for tree 1, tree 2, and tree 3. This stage involves an iterative procedure with convergence criteria based on two conditions: first, if the difference in mean square error (MSE) between two consecutive iterations falls below a predefined threshold, and second, if the maximum allowed number of iterations is reached. Following the selection stage, the estimation stage is carried out using maximum likelihood estimation and Restricted Maximum Likelihood (REML) methods. The 3Trees-EvTree and 3Trees-CTree algorithms are structured similarly to Algorithm 1, with the primary distinction being in the tree selection step.

**Algorithm 1:** Alg-3Trees.

### CART algorithm

The CART algorithm, introduced by Breiman [25], is one of the most widely used regression tree approaches in recent years. This method is based on a binary splitting process, performed recursively using predictor variables to generate terminal nodes that contain predictions for the target outcome. Each variable in the dataset is divided into distinct points, followed by the computation of the sum of squared errors or the MSE. The sum of squared errors is calculated separately for each partition and then summed. The splitting point that results in the smallest sum of squared errors is selected as the splitting point to be used as the root node. Given a dataset $D=\{\left(x_{i},y_{i}\right)\},\;i=1,2,\ldots ,N$ with $x_{i}\in R^{d}$ and $y_{i}\in R^{K}$, the squared error R(t) of the node t is defined as follows (Eq. (3)):

(3) $$R\left(t\right)=\frac{1}{N}\sum_{x_{i}\in t}{\left(y_{i}-\bar{y}\left(t\right)\right)}^{2}$$

for each node t, $\bar{y}\left(t\right)$ is formulated as $\bar{y}\left(t\right)=\frac{1}{N(t)}\sum_{x_{i}\in t}y_{i}$. Furthermore, if s is defined as a split of a node t into $t_{L}$ and $t_{R}$, then the split s is selected by maximizing R(s,t) of the cost by s with following function (Eq. (4)):

(4) $$\Delta R\left(t\right)=\left(s,t\right)=R\left(t\right)-R\left(t_{L}\right)-R\left(t_{R}\right)$$

The CART algorithm is briefly outlined in pseudocode form in Algorithm 2 [17].

**Algorithm 2:** Regression trees.

To avoid excessive splitting that may lead to increasingly complex trees and result in overfitting, the CART method incorporates a pruning process. Decision tree pruning uses cost complexity, which aims to find the optimal balance between prediction accuracy and model complexity, thereby producing a model that generalizes well to new data. The cost complexity equation $R_{\alpha }\left(T\right)$, with *T* denoting the number of trees and α≥0, is given by the following equation (Eq. (5)):

(5) $$R_{\alpha }\left(T\right)=R\left(T\right)+\alpha \left|T\right|$$

### CTree algorithm

The CTree algorithm is a machine learning technique that employs robust statistical testing (significance testing) to construct regression trees, resulting in more reliable and well-generalized models [24,26]. The significance test involves the permutation of variables to determine whether the splits at each step of the decision tree construction are meaningful, facilitating unbiased recursive partitioning of predictors [24,27]. The purpose of this process is to ensure that each split made within the decision tree significantly enhances the tree's ability to predict the target variable based on the permutation of target variable values. If a split does not provide significant improvement, it is deemed unimportant and not performed.

The CTree algorithm for selecting trees $T_{1},T_{2}$ and $T_{3}$ involves the following stages:

1. Let $Y_{ij}$ be the response variable vector consisting of $n_{total}$ observations, and suppose $X_{ij}$ and $Z_{j}$ be the predictor vector with $n_{total}\times p_{1}$ and $n_{\text{total}}\times p_{2}$ dimensions, respectively.
2. Evaluating a potential split is conducted through a hypothesis test, where the null hypothesis, $H_{0}:P\left(Y_{ij}|X_{ij}\right)=P\left(Y_{ij}\right),\;H_{0}:P\left(Y_{ij}|Z_{j}\right)=P\left(Y_{ij}\right),\;H_{0}:P\left(Y_{ij}|X_{ij},Z_{j}\right)=P\left(Y_{ij}\right)$ asserts that no relationship exists between the predictor and the response variables. This hypothesis is assessed by examining a set of partial null hypotheses. If the null hypothesis is rejected, it indicates a statistically significant association between the predictor and the response, justifying the split. Conversely, if the null hypothesis cannot be rejected, the split is deemed unnecessary and is not performed.
3. Calculating the p-value for each partial null hypothesis. The p-value is a statistical measure that indicates the strength of the evidence against the null hypothesis. If the minimum p-value computed is lower than the predefined significance α level, the global null hypothesis is rejected, and a split is made at the current node. Otherwise, no further splitting occurs, or the recursive process stops, and the node is designated as a terminal node.
4. Determining the threshold for the minimum p-value is based on adjusting for multiple comparisons while maintaining a fixed significance level, α. At this stage, the Bonferroni method can be applied, where the significance level is divided by the number of comparisons being made.
5. Selecting the variable for the splitting process involves evaluating the appropriate t-statistic under the null hypothesis, $H_{0}$. If a split is to be made, the variable to be split is chosen based on the t-statistic corresponding to the null hypothesis $H_{0}$. This same t-statistic is also used to calculate the p-value necessary for assessing the significance of $H_{0}$.

The CTree algorithm can be summarized in pseudocode form as follows in Algorithm 3 [17].

**Algorithm 3:** Conditional Inference Regression tree.

### EvTree algorithm

The EvTree method, developed for CART method, is an evolutionary algorithm aimed at finding globally optimal tree structures rather than the locally optimal solutions typically generated by traditional methods such as CART and C4.5. This approach leverages evolutionary concepts such as mutation, crossover, and selection to refine a population of candidate trees, optimizing them according to a fitness function that balances prediction accuracy and model simplicity. Unlike conventional tree models, which use a greedy algorithm to optimize each split individually, EvTree conducts a broader, more comprehensive search over the parameter space, resulting in models that are not only more accurate but often simpler and more efficient [23].

Step-by-step process of the EvTree algorithm:

1. *Initialization*: The process starts with generating a population of random trees. Each tree is initialized by assigning a valid split rule at the root node. If a valid split cannot be generated, the process is repeated until all trees in the population are initialized.
2. *Parent Selection*: In each iteration, trees are selected as ``parents'' to undergo modification. If the crossover operation is selected, two parents are chosen randomly for recombination.
3. *Variation Operators*: The evolutionary process relies on several variation operators (see Fig. 1):
  a) *Split Mutation*: A random terminal node is selected, and a new split rule is applied, converting it into an internal node.
  b) *Prune Mutation*: A random internal node is selected and pruned, reducing the complexity of the tree.
  c) *Major Split Rule Mutation*: This changes both the splitting variable and the split point at a given internal node.
  d) *Minor Split Rule Mutation*: This operator makes small adjustments to the split point without changing the splitting variable.
  e) *Crossover*: Subtrees from two-parent trees are exchanged to create new trees.

**Fig. 1:**
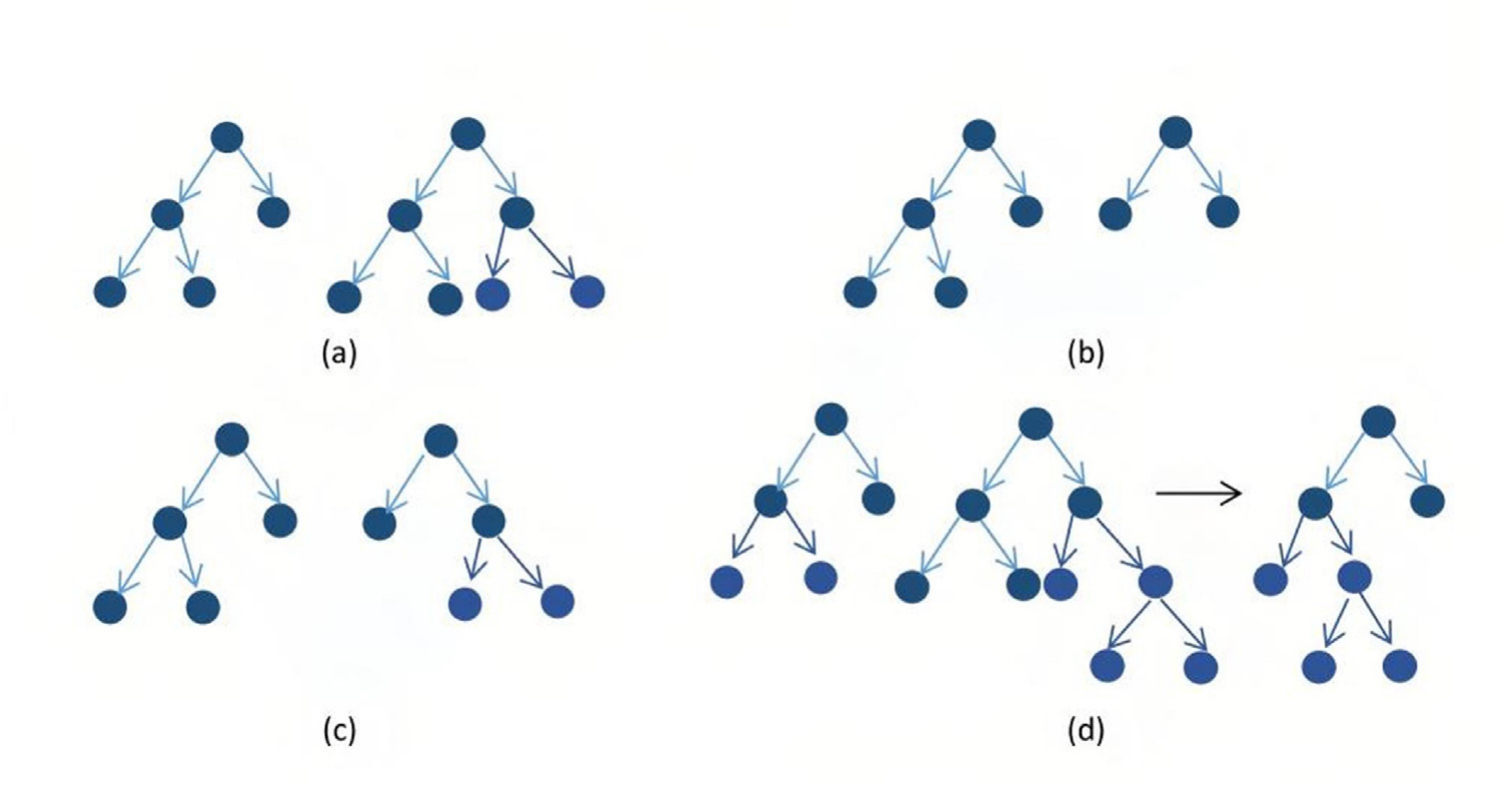
Variation operators of Evtree algorithm (a) Grow (b) Prune (c) Mutation (d) Crossover.
4. *Evaluation Function*: The quality of each tree is assessed using an evaluation function that balances prediction accuracy (e.g., misclassification rate or MSE) and complexity (e.g., the number of terminal nodes). Trees with better performance are favored in the next generation.
5. *Survivor Selection*: After each iteration, a competition occurs between parent trees and their offspring. The tree with the better evaluation score is retained, ensuring the population evolves towards better solutions.
6. *Termination*: The algorithm terminates when the quality of the best solutions stabilizes over several iterations or after a predefined number of iterations. The best-performing tree is then selected as the final solution.

The EvTree algorithm is briefly outlined in pseudocode in Algorithm 4 [17].

**Algorithm 4:** Evtree.

## Method validation

### Simulation study design

In this section, simulation studies are perfomed to evaluate the performance of the 3Trees using proposed algorithms, CTree and EvTree algorithm, to estimate the tree component in comparison with the previous algorithm. The different simulated datasets involve hierarchical mixed-effects models under three functional forms of response variable: linear without interaction (M1), semilinear with interaction (M2), and nonlinear functions (M3) with the response variable across 12 distinct settings. These models are derived from the tree-based mixed-effects framework outlined by Gottard et al. [19]. The detailed simulation design is provided in Table 1. Random effects were restricted to random intercepts. Predictors at both level 1 and level 2 were generated following normal and binomial distributions to assess the optimization of the proposed tree-based approach, particularly regarding the unbiased recursive partitioning of mixed-scale variables. Both the fixed-effect parameters and regression tree components, including *Intraclass Correlation Coefficient* (ICC) values and covariate correlations, were varied. The complete set of fixed and random effect parameters is also outlined in Table 1.

**Table 1:** Simulation parameters for fixed and random effects in the simulation settings.

Scenario	Type of Model	$\beta_{0}$	$\beta_{1}$	$\beta_{2}$	$\beta_{3}$	$\beta_{4}$	$\gamma_{1}$	$\gamma_{2}$	$\gamma_{3}$	$\mu_{1}$	$\mu_{2}$	$\mu_{3}$	ICC	$\sigma_{u}^{2}$	$\sigma_{\varepsilon }^{2}$	Correlation	ρ
M1	Linear	5	2	0	3	1	3	0	1	0	0	0	high	3	1	high	0.8
	Linear	5	2	0	3	1	3	0	1	0	0	0	small	0.3	0.5	high	0.8
	Linear	5	2	0	3	1	3	0	1	0	0	0	high	3	1	small	0.2
	Linear	5	2	0	3	1	3	0	1	0	0	0	small	0.3	0.5	small	0.2
M2	Semilinear+ Interaction	5	2	0	3	1	2	0	1	3	2	−2	high	3	1	high	0.8
	Semilinear+ Interaction	5	2	0	3	1	2	0	1	3	2	−2	small	0.3	0.5	high	0.8
	Semilinear+ Interaction	5	2	0	3	1	2	0	1	3	2	−2	high	3	1	small	0.2
	Semilinear+ Interaction	5	2	0	3	1	2	0	1	3	2	−2	small	0.3	0.5	small	0.2
M3	Nonlinear	5	2	0	2	2	3	0	2	2	1	0	high	3	1	high	0.8
	Nonlinear	5	2	0	2	2	3	0	2	2	1	0	small	0.3	0.5	high	0.8
	Nonlinear	5	2	0	2	2	3	0	2	2	1	0	high	3	1	small	0.2
	Nonlinear	5	2	0	2	2	3	0	2	2	1	0	small	0.3	0.5	small	0.2

For each simulation dataset, the data-generating process is as follows. Four random variables are first generated for level 1 and there are three random variables for level 2 to be considered. These variables are denoted as vectors $X={\left[X_{1},X_{2},X_{3},\;X_{4}\right]}^{T}$ and $Z={\left[Z_{1},Z_{2},Z_{3}\right]}^{T}$, respectively. Five variables $X_{1},X_{2},X_{3},Z_{1},Z_{2}$ are constructed to the following multivariate distribution.

X∼MN(0,Σ),Z∼MN(0,Γ)

Note that two structures of covariance matrix for each level covariate are considered. Previously, we set two specifications of correlation, including ρ=0.2 (small) and ρ=0.8 (high). The covariance matrices are defined as follow:

$$\Sigma_{\text{low}}=\left(\begin{array}{ccc} 1 & 0.2 & 0.2\\ 0.2 & 1 & 0.2\\ 0.2 & 0.2 & 1 \end{array}\right),\;\Gamma_{low}=\left(\begin{array}{cc} 1 & 0.2\\ 0.2 & 1 \end{array}\right),\;\text{and}\;\Sigma_{\text{high}}=\left(\begin{array}{ccc} 1 & 0.8 & 0.8\\ 0.8 & 1 & 0.8\\ 0.8 & 0.8 & 1 \end{array}\right),\;\Gamma_{\text{high}}=\left(\begin{array}{cc} 1 & 0.8\\ 0.8 & 1 \end{array}\right)$$

Additionally, two variables, $X_{4}$ and $Z_{3}$, are generated based on binary variables as follow:

$$X_{4}∼bin\left(n,0.5\right),\;Z_{3}∼bin\left(n,0.5\right)$$

In the following simulations, the random components, between and within cluster variance, are drawn from the following normal distributions, denoted as $u_{j}∼N\left(0,\;\sigma_{u}^{2}\right)$ and $\varepsilon_{ij}∼N\left(0,\sigma_{\varepsilon }^{2}\right)$ respectively. To obtain ICC values of 0.75 and 0.375, $\sigma_{u}^{2}=3,\;\sigma_{\varepsilon }^{2}=1$ and $\sigma_{u}^{2}=0.3,\;\sigma_{\varepsilon }^{2}=0.5$ are determined. Next, we generate a response variable $\left(Y_{ij}\right)$ based on the above information under three model scenarios following equations:

a) Scenario M1: linear mixed-effect model
  (6) $$Y_{ij}=\beta_{0}+\beta_{1}X_{1ij}+\beta_{2}X_{2ij}+\beta_{3}X_{3ij}+\beta_{4}X_{4ij}+\gamma_{1}Z_{1j}+\gamma_{2}Z_{2j}+\gamma_{3}Z_{3j}+u_{j}+\varepsilon_{ij}$$
b) Scenario M2: semilinear mixed-effect model including interaction term
  (7) $$\begin{array}{ccc} Y_{ij} & = & \beta_{0}+\beta_{1}X_{1ij}+\beta_{2}X_{2ij}+\beta_{3}X_{3ij}+\beta_{4}X_{4ij}+\gamma_{1}Z_{1j}+\gamma_{2}Z_{2j}+\gamma_{3}Z_{3j}+\mu_{1}I\left(X_{1ij}>0\right)+\mu_{2}I\left(Z_{3j}>0\right)\\ & & +\;\mu_{3}I\left(X_{1ij}>0\right)I\left(Z_{3j}>0\right)+u_{j}+\varepsilon_{ij} \end{array}$$
c) Scenario M3: Nonlinear mixed-effect model
  (8) $$Y_{ij}=\beta_{0}+\beta_{1}X_{1ij}+\beta_{2}X_{2ij}+\beta_{3}X_{3ij}+\beta_{4}X_{4ij}+\gamma_{1}Z_{1j}+\gamma_{2}Z_{2j}+\gamma_{3}Z_{3j}+\mu_{1}X_{1ij}^{2}+\mu_{2}Z_{2ij}ln\left|X_{3ij}\right|+u_{j}+\varepsilon_{ij}$$

Note that true parameters for all model scenarios are given in Table 1. We also set two parameters at both level 1 and level 2 to zero, or considered them negligible, to examine the algorithm's ability to handle irrelevant predictors or noise variables.

A hierarchical structure of j=70clusters with $n_{j}=50$ observations each is randomly generated, resulting in a total of 3500 observations $\left(n_{total}=3500\right)$ for M1, M2, and M3 each. Moreover, to investigate the consistency of the model in estimating parameters and comparing the prediction accuracy, several datasets of sizes $n_{total}=10000,\;5000,\;3500,\;1500,\;750$ are generated to test the model under scenario M2, where both the correlation and ICC are chosen to high levels. For $n_{total}=3500$, the data are splitted into training and testing sets with a 50 % ratio, resulting in j=35 clusters, where each cluster contains $n_{j}=25$ observations. Thus, the total number of observations for both the training and testing sets is $n_{total}=1750$ observations each. This procedure is applied to the other sample sizes. The following step is repeated for 100 times to create 100 datasets.

For fitting each simulation settings, we run six different models, including our proposed models, 3Trees-CTree and 3Trees-EvTree, 3Trees-CART [19] with two distincts of complexity parameters, and a linear mixed-effect model. 3Trees-CART models, proposed by Gottard et al. [19], are fitted R *Packages rpart* [28] for the tree component and *lme4* for the linear mixed-effect component of Eq. (1). The *rpart* function in this package implements the recursive partitioning method based on CART algorithm. All tuning parameters for rpart function are set using their default values, except for complexity parameter with cp=0 and cp=0.0001, *maxdepth*=3, and *nmin*=10. Meanwhile, Packages *party* [29] estimated for tree part in 3Trees-CTree. The *ctree* function deploys a conditional inference tree algorithm that recursively partitions the data based on statistically significant associations between covariates and the response variable, while controlling for overfitting through hypothesis testing. Only *maxdepth*=3 is adjusted, while all other hyperparameters are left at their default settings. This function is also available in the *partykit* package [30], allowing for the inclusion of this analysis, to which the 3Trees-TSCTree model refers. Then, the *evtree* package was used for the 3Trees-EvTree model. This package provides an implementation of evolutionary learning for constructing globally optimal CART in R. Therefore, the tree maximum depth of *evtree* function is 3 for tuning, while the default settings are preserved for all other turning parameters. For the linear mixed model, we utilized the *lmer* function available in the *lme4* package.

The model performances are assessed with following, i) Absolute bias average-the difference between the mean of estimated parameters and true parameters. ii) For predictive performance, these models are compared in terms of MSE and Predictive MSE (PMSE). This refers to predictions that consider only the fixed-effects component. Meanwhile, clusMSE and clusPMSE are criteria that refer to the prediction results involving both the fixed and random effects. All criteria formulation may be written as (Eq. (9)):

(9) $$MSE=PMSE=ClusMSE=ClusPMSE=\frac{1}{n_{total}}\sum_{i=1}^{n_{j}}\sum_{j=1}^{J}{\left(y_{ij}-{\hat{y}}_{ij}\right)}^{2}$$

### Results of simulation studies

In our comparative analysis of the 3Trees (CART, EvTree, CTree, TSCTree) and LMMs algorithms, we ran 100 times to ensure robustness in our findings. First, the performance of the methods are evaluated in terms of MSE, PMSE, clusMSE, and clusPMSE. The averages of MSE are summarized in Table 2, Table 3, along with their standard errors in parentheses for the methods being compared. The left part portrays the results corresponding to high ICC values, while the right one illustrates the results for low ICC values. These tables differ based on the varied correlation values. The bold type values represent the smallest error rates for each scenario. As shown in the first and second tables, the prediction results of models including random effects generally outperform those without random effects in both training and testing data for all the information scenarios. From these tables, it is remarkable that the clusMSE and clusPMSE values are smaller than MSE and PMSE. This confirms that the constructed simulation model conforms exactly with the expected specifications. Furthermore, all 3Trees models, including the LMMs, display relatively higher MSE values under fully nonlinear conditions (scenario M3). This finding is consistent with Gottard et al. [19], who suggests that as the complexity of the function increases, such as with nonlinear term functions, a greater maximum depth is required to reduce the MSE. Also, since the base model is a linear mixed-effects model, these models may not be suitable for such conditions and may fail to capture the true nature of the data. This problem has been highlighted by Blozis and Harring [31].

**Table 2:** Results of the 100 simulation runs in terms of the averages and standard deviations of MSE, ClusMSE, PMSE dan ClusPMSE for six models. The smallest are marked in bold.

ρ*=0.8, ICC=0.75*					ρ*=0.8, ICC=0.375*				
	MSE	ClusMSE	PMSE	ClusPMSE		MSE	ClusMSE	PMSE	ClusPMSE
**Scenario M1**					**Scenario M1**				
3Trees-CART (cp=0.0001)	3.569 (0.608)	0.914 (0.029)	3.674 (0.592)	1.086 (0.040)	3Trees-CART (cp=0.0001)	6.747 (0.479)	**4.604** (0.136)	7.393 (0.529)	5.451 (0.195)
3Trees-CART (cp=0)	**3.150** (0.469)	**0.913** (0.03)	**3.264** (0.460)	1.087 (0.041)	3Trees-CART (cp=0)	**6.725** (0.462)	**4.604** (0.137)	7.355 (0.515)	5.451 (0.192)
3Trees-CTree	3.235 (0.420)	0.946 (0.029)	3.305 (0.405)	1.050 (0.039)	3Trees-CTree	7.059 (0.453)	4.767 (0.137)	**7.340** (0.484)	5.272 (0.187)
3Trees-EvTree	3.639 (0.487)	0.946 (0.029)	3.673 (0.477)	1.051 (0.040)	3Trees-EvTree	7.478 (0.508)	4.780 (0.139)	7.652 (0.537)	5.250 (0.183)
LMMs	3.808 (0.492)	0.954 (0.029)	3.862 (0.487)	**1.040** (0.039)	LMMs	7.696 (0.523)	4.818 (0.140)	7.780 (0.531)	**5.202** (0.184)
3Trees-TSCTree	3.860 (0.502)	0.954 (0.029)	3.883 (0.489)	**1.040** (0.039)	3Trees-TSCTree	7.765 (0.551)	4.820 (0.140)	7.810 (0.549)	**5.202** (0.184)
**Scenario M2**					**Scenario M2**				
3Trees-CART (cp=0.0001)	3.774 (0.846)	1.153 (0.373)	3.865 (0.839)	1.338 (0.430)	3Trees-CART (cp=0.0001)	7.384 (0.729)	4.965 (0.452)	7.938 (0.759)	5.791 (0.492)
3Trees-CART (cp=0)	**3.683** (0.813)	1.189 (0.381)	3.781 (0.834)	1.379 (0.444)	3Trees-CART (cp=0)	**7.353** (0.742)	4.967 (0.451)	7.917 (0.784)	5.792 (0.489)
3Trees-CTree	5.107 (0.874)	1.878 (0.104)	5.207 (0.891)	2.076 (0.112)	3Trees-CTree	9.048 (0.908)	5.670 (0.204)	9.365 (0.874)	6.288 (0.217)
3Trees-EvTree	3.689 (0.532)	**0.944** (0.032)	**3.707** (0.524)	**1.064** (0.042)	3Trees-EvTree	7.638 (0.503)	**4.760** (0.156)	**7.830** (0.556)	**5.270** (0.180)
LMMs	7.443 (0.779)	3.296 (0.178)	7.485 (0.759)	3.575 (0.172)	LMMs	11.479 (0.788)	7.143 (0.294)	11.619 (0.775)	7.756 (0.314)
3Trees-TSCTree	7.510 (0.780)	3.296 (0.178)	7.505 (0.750)	3.575 (0.172)	3Trees-TSCTree	11.561 (0.790)	7.145 (0.294)	11.655 (0.773)	7.756 (0.314)
**Scenario M3**					**Scenario M3**				
3Trees-CART (cp=0.0001)	4.808 (0.599)	2.269 (0.263)	5.169 (0.579)	2.805 (0.299)	3Trees-CART (cp=0.0001)	8.362 (0.590)	5.894 (0.296)	9.201 (0.727)	7.115 (0.440)
3Trees-CART (cp=0)	**4.760** (0.593)	2.271 (0.265)	**5.122** (0.587)	2.806 (0.300)	3Trees-CART (cp=0)	**8.348** (0.579)	5.890 (0.297)	**9.200** (0.724)	7.119 (0.443)
3Trees-CTree	6.539 (0.716)	4.130 (0.589)	7.036 (0.715)	4.767 (0.621)	3Trees-CTree	10.448 (0.74)	7.960 (0.543)	11.262 (0.909)	9.193 (0.727)
3Trees-EvTree	4.768 (0.548)	**1.876** (0.194)	5.239 (0.531)	**2.505** (0.227)	3Trees-EvTree	8.820 (0.602)	**5.818** (0.305)	9.620 (0.711)	**7.099** (0.382)
LMMs	12.985 (1.057)	9.800 (0.878)	13.152 (0.974)	10.64 (0.812)	LMMs	16.819 (1.069)	13.528 (0.873)	17.28 (1.220)	14.858 (0.985)
3Trees-TSCTree	13.041 (1.068)	9.807 (0.879)	13.163 (0.968)	10.639 (0.811)	3Trees-TSCTree	16.882 (1.074)	13.539 (0.874)	17.29 (1.219)	14.856 (0.987)

**Table 3:** Results of the 100 simulation runs in terms of the averages and standard deviations of MSE, ClusMSE, PMSE dan ClusPMSE under six models. The smallest are marked in bold (Continue).

ρ*=0.2, ICC=0.75*					ρ*=0.2, ICC=0.375*				
	MSE	ClusMSE	PMSE	ClusPMSE		MSE	ClusMSE	PMSE	ClusPMSE
**Scenario M1**					**Scenario M1**				
3Trees-CART (cp=0.0001)	3.537 (0.548)	0.913 (0.034)	3.633 (0.540)	1.089 (0.040)	3Trees-CART (cp=0.0001)	6.811 (0.481)	4.597 (0.154)	7.363 (0.480)	5.421 (0.190)
3Trees-CART (cp=0)	**3.141** (0.442)	**0.912** (0.034)	3.247 (0.433)	1.088 (0.039)	3Trees-CART (cp=0)	**6.778** (0.459)	**4.596** (0.153)	7.347 (0.476)	5.424 (0.194)
3Trees-CTree	3.164 (0.410)	0.947 (0.034)	**3.211** (0.406)	1.052 (0.039)	3Trees-CTree	7.060 (0.444)	4.748 (0.161)	**7.328** (0.440)	5.262 (0.173)
3Trees-EvTree	3.598 (0.462)	0.947 (0.035)	3.618 (0.459)	1.051 (0.037)	3Trees-EvTree	7.526 (0.484)	4.760 (0.162)	7.675 (0.496)	5.238 (0.173)
LMMs	3.755 (0.488)	0.954 (0.034)	3.779 (0.498)	**1.042** (0.037)	LMMs	7.747 (0.491)	4.797 (0.164)	7.835 (0.514)	5.198 (0.170)
3Trees-TSCTree	3.812 (0.49)	0.954 (0.034)	3.812 (0.495)	**1.042** (0.037)	3Trees-TSCTree	7.797 (0.489)	4.799 (0.164)	7.851 (0.509)	**5.197** (0.170)
**Scenario M2**					**Scenario M2**				
3Trees-CART (cp=0.0001)	4.326 (1.037)	1.413 (0.432)	4.466 (1.018)	1.642 (0.492)	3Trees-CART (cp=0.0001)	7.930 (0.936)	5.285 (0.426)	8.542 (0.944)	6.221 (0.523)
3Trees-CART (cp=0)	4.216 (1.066)	1.392 (0.432)	4.362 (1.060)	1.619 (0.493)	3Trees-CART (cp=0)	7.913 (0.946)	5.285 (0.421)	8.536 (0.960)	6.228 (0.517)
3Trees-CTree	3.780 (0.730)	1.350 (0.389)	3.890 (0.743)	1.507 (0.415)	3Trees-CTree	7.768 (0.645)	5.182 (0.405)	8.072 (0.694)	5.786 (0.450)
3Trees-EvTree	**3.758** (0.561)	**0.947** (0.034)	**3.825** (0.540)	**1.071** (0.045)	3Trees-EvTree	**7.732** (0.591)	**4.780** (0.163)	**7.867** (0.613)	**5.305** (0.180)
LMMs	7.594 (0.769)	3.303 (0.168)	7.661 (0.813)	3.589 (0.214)	LMMs	11.575 (0.832)	7.152 (0.287)	11.721 (0.803)	7.779 (0.326)
3Trees-TSCTree	7.676 (0.791)	3.304 (0.168)	7.701 (0.825)	3.589 (0.214)	3Trees-TSCTree	11.653 (0.856)	7.154 (0.288)	11.75 (0.813)	7.779 (0.326)
**Scenario M3**					**Scenario M3**				
3Trees-CART (cp=0.0001)	4.799 (0.645)	2.324 (0.254)	5.145 (0.678)	2.820 (0.312)	3Trees-CART (cp=0.0001)	**8.464** (0.621)	6.002 (0.325)	**9.322** (0.626)	7.188 (0.364)
3Trees-CART (cp=0)	4.786 (0.638)	2.324 (0.254)	**5.130** (0.674)	2.819 (0.313)	3Trees-CART (cp=0)	8.466 (0.634)	6.002 (0.325)	9.340 (0.622)	7.192 (0.363)
3Trees-CTree	7.436 (0.866)	5.088 (0.763)	7.801 (0.887)	5.676 (0.735)	3Trees-CTree	11.156 (0.891)	8.736 (0.673)	12.166 (0.918)	10.098 (0.726)
3Trees-EvTree	**4.769** (0.604)	**1.925** (0.174)	5.198 (0.624)	**2.539** (0.208)	3Trees-EvTree	8.792 (0.623)	**5.872** (0.282)	9.683 (0.614)	**7.150** (0.352)
LMMs	13.042 (1.003)	9.922 (0.788)	12.958 (1.01)	10.537 (0.759)	LMMs	16.859 (0.906)	13.642 (0.68)	17.261 (1.173)	14.855 (0.99)
3Trees-TSCTree	13.114 (1.016)	9.929 (0.789)	12.982 (1.019)	10.536 (0.760)	3Trees-TSCTree	16.924 (0.911)	13.654 (0.681)	17.284 (1.158)	14.854 (0.99)

According to the ICC value, as a result, the prediction of all 3Trees and LMMs models significantly performs well when the random effect is relatively large, despite the different (small and high) correlation considered. In contrast, an increase in error prediction values is observed for all corresponding methods when the ICC is low across all criteria. This situation implies that the smaller the variance between groups, the more difficult it is for the all models to predict accurately the response variable. Meanwhile, the MSE values do not manifest a significant difference between small (ρ=0.2) and high (ρ=0.8) correlation for all compared methods, suggesting that the 3Trees models may not adequately account for the importance of correlation in its predictions. This finding corresponds to Gottard's study on the 3Trees model, which demonstrated that the 3Trees algorithm remains unaffected by the correlation between predictors. This showcases the robustness of the 3Trees approach in addressing multicollinearity, making it especially effective in scenarios involving highly correlated variables.

With regards to a linear function (scenario M1), on average, 3Trees-CART outperforms compared with the other models when the complexity parameter is set to zero (cp=0), except for PMSE and clusPMSE criteria, where LMMs and 3Trees-TSCTree models perform slightly better. However, 3Trees-CART performs more effectively in predicting the response variable compared to LMMs and 3Trees-TSCTree for the M1 and M2 scenarios. In Table 1, Table 2, notice that if the true underlying model is semilinear (scenario M2) and nonlinear (scenario M3), as expected, it is clear that the EvTree algorithm performs better than alternatives in terms of clusMSE and clusPMSE. Surprisingly, our proposed method (EvTree) achieves superior performance in all evaluated criteria when the correlation among explanatory variables is low. The findings reveal that the evtree algorithm performs optimally in selecting trees within the 3Trees framework, minimizing prediction errors. However, this method does not appear to perform better than the previous method when the scenario involves a linear function (scenario M1).

The other proposed algorithm, 3Trees-CTree stands out after 3Trees-EvTree for M2 scenario when the correlation among covariates is small. However, this method shows no marked improvement in the remaining scenarios. On average, LMMs and 3Trees-TSCTree perform rather poorly, except for the cases in PMSE criteria for scenario M1. Moreover, in the non-linear model of scenario M3, these models show MSE values that are substantially higher than those of the other models. Note that the performance of the 3Trees-TSCTree model is notably inferior compared to other models in the 3Trees framework, positioning closely with results from the LMMs model. This similarity arises mainly because the TSCTree's node predictions tend to produce a singular fit matrix, a result of collinearity among the invariant structures within the nodes.

**Fig. 2:**
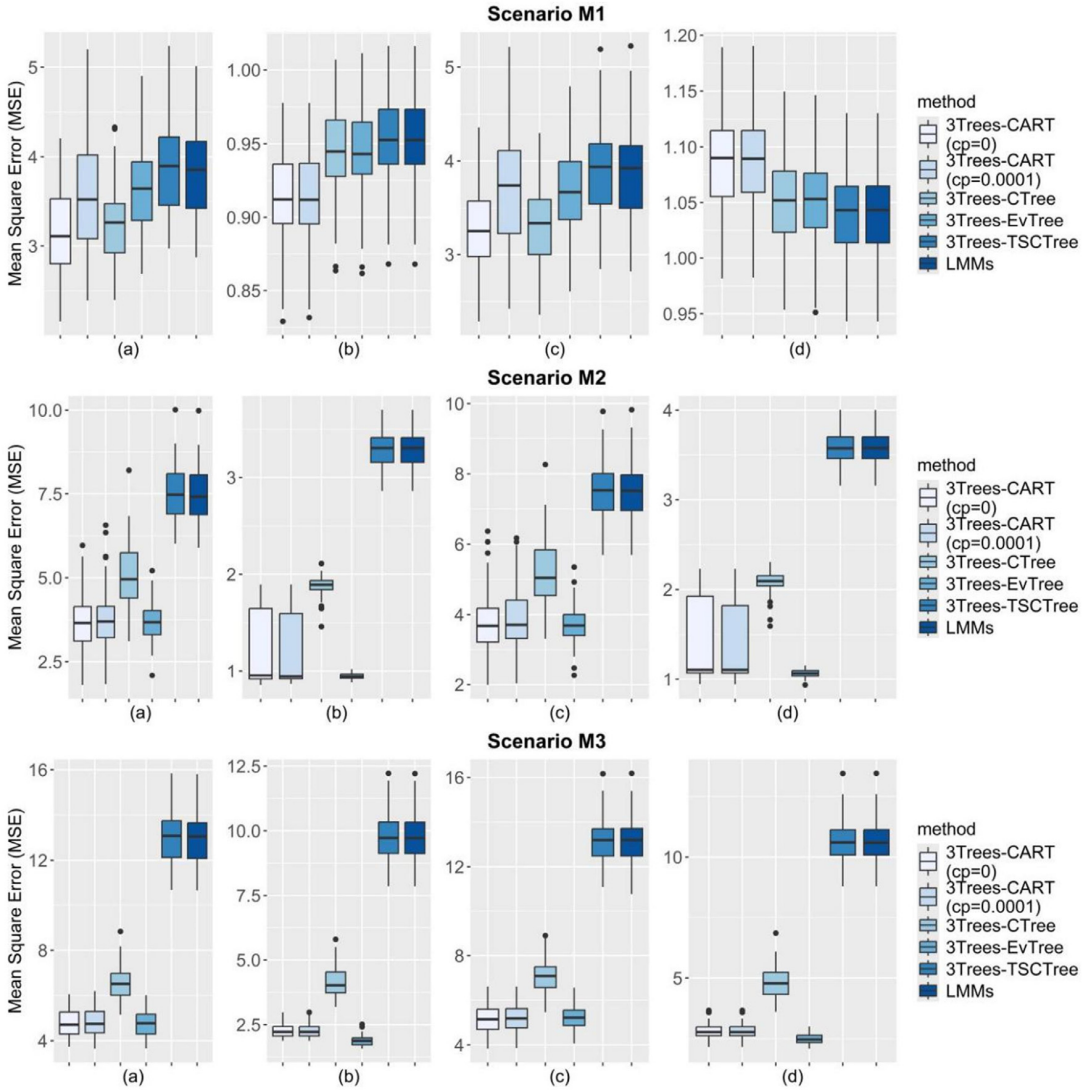
Boxplots for distributions of evaluation metrics (a) MSE (b) ClusMSE (c) PMSE (d) ClusPMSE.

Boxplots of error predictions for ρ=0.8 and ICC=0.75 are also displayed in Fig. 2. These boxplots are constructed to depict the distribution of error prediction values over 100 repetitions. In this figure, each graph (Fig. 2a-2d) contains six boxplots for prediction accuracy for the three scenarios. From this plot, we can easily see the performance of our proposed methods with respect to all criteria. Within scenario M1, all compared models have nearly the same variability. Simultaneously, in relation to scenario M2, the 3Trees-EvTree and 3Trees-CTree methods are expected to show shorter boxplots with lower medians considering clusMSE and clusPMSE criteria. Their distributions are narrow, suggesting they provide more stable and consistent predictions. The 3Trees-EvTree model, in particular, has the lowest error prediction median, making it the best performer overall. At once, the 3Trees-CART method exposes high variability, with a wide range of these criteria across the simulations. However, this method shows lower variability than 3Trees-CTree based on scenario M2. In contrast, EvTree produces consistent results. Furthermore, as observed, there is almost no difference in error predictions for LMMs and 3Trees-TSCTree across the M1, M2, and M3 scenarios. Note that boxplots for additional variations in correlation values and ICC levels are not included in this paper because these boxplots do not differ significantly from those obtained previously.

A further consideration is the estimation of parameters in the linear component both level-1 $\left(\beta_{0},\beta_{1},\beta_{2},\beta_{3},\beta_{4}\right)$ and level-2 $\left(\gamma_{1},\gamma_{2},\gamma_{3}\right)$ Table 4 summarizes the results of various statistics obtained from 100 runs with each of the methods used, considering ρ=0.8 and ICC=0.75. This table contains the mean, standard deviation (in parentheses) and bias. According to scenario M1, it is observed that the corresponding biases of the linear component parameters for all methods are very small as all the means of parameters were similar to the true value for almost all parameters. LMMs and 3Trees-TSCTree have the highest number of parameters with the slightest bias. At the same time, 3Trees-EvTree also estimates these parameters quite accurately. However, the 3Trees-CTree method frequently overestimates several parameters such as $\beta_{0}$, $\gamma_{1}$, and $\gamma_{3}$, while the 3Trees-CART (cp=0 and cp=0.0001) method reveals bias only in the $\beta_{0}$ parameter. As part of scenario M2, most methods appear to slightly over- or underestimate $\beta_{0}$, $\beta_{1}$ and $\gamma_{1}$, stand out with significant bias in most parameters, excluding $\beta_{1}$. Following scenario M1, all parameters can also be estimated with low bias in most of the settings throughout scenario M3. However, the 3Trees-CTree method shows bias in nearly all of its parameter estimates. Similar results are shown in other settings (Table 10–Appendix B).

**Table 4:** Parameter averages, standard deviations and bias of estimated fixed-effect components produced by 3Trees and LMMs (ρ=0.8 and ICC=0.75).

Parameter																
	$\beta_{0}$	Bias	$\beta_{1}$	Bias	$\beta_{2}$	Bias	$\beta_{3}$	Bias	$\beta_{4}$	Bias	$\gamma_{1}$	Bias	$\gamma_{2}$	Bias	$\gamma_{3}$	Bias
**Scenario M1**																
True Value	5.000	–	2.000	–	0.000	–	3.000	–	1.000	–	3.000	–	0.000	–	1.000	–
3Trees-CART (cp=0.0001)	2.728 (0.748)	2.272	2.003 (0.054)	−0.003	−0.008 (0.054)	0.008	3.002 (0.053)	**−0.002**	0.990 (0.048)	0.010	3.014 (0.379)	**−0.014**	−0.047 (0.373)	0.047	1.033 (0.423)	**−0.033**
3Trees-CART (cp=0)	1.123 (1.343)	3.877	1.997 (0.054)	0.003	−0.006 (0.054)	0.006	3.006 (0.054)	−0.006	0.992 (0.048)	**0.008**	2.943 (0.425)	0.057	−0.038 (0.419)	0.038	1.057 (0.419)	−0.057
3Trees-CTree	0.844 (1.478)	4.156	1.961 (0.063)	0.039	0.001 (0.045)	**−0.001**	2.917 (0.084)	0.083	0.990(0.049)	0.010	1.164 (0.656)	1.836	−0.067 (0.328)	0.067	0.592 (0.543)	0.408
3Trees-EvTree	4.932 (0.706)	0.068	2.002 (0.047)	−0.002	0.001 (0.047)	**−0.001**	2.995 (0.048)	0.005	0.992 (0.048)	**0.008**	2.984 (0.354)	0.016	−0.029 (0.348)	0.029	1.041 (0.402)	−0.041
LMMs	4.987 (0.483)	**0.013**	2.001 (0.045)	**−0.001**	0.001 (0.045)	**−0.001**	2.996 (0.045)	0.004	0.991 (0.049)	0.009	3.019 (0.363)	−0.019	−0.022 (0.358)	0.022	1.045 (0.432)	−0.045
3Trees-TSCTree	4.986 (0.293)	0.014	2.001 (0.045)	**−0.001**	0.001 (0.045)	**−0.001**	2.996 (0.045)	0.004	0.991 (0.049)	0.009	3.014 (0.349)	**−0.014**	−0.02 (0.345)	**0.020**	1.048 (0.416)	−0.048
**Scenario M2**																
True Value	5.000	–	2.000	–	0.000	–	3.000	–	1.000	–	2.000	–	0.000	–	1.000	–
3Trees-CART (cp=0.0001)	0.981 (1.303)	4.019	2.619 (0.139)	−0.619	0.025 (0.052)	−0.025	3.015 (0.051)	−0.015	1.002 (0.053)	**−0.002**	2.086 (0.446)	−0.086	−0.656 (0.55)	0.656	0.945 (0.466)	0.055
3Trees-CART (cp=0)	0.409 (1.439)	4.591	2.625 (0.143)	−0.625	0.023 (0.053)	−0.023	3.016 (0.052)	−0.016	1.003 (0.054)	−0.003	2.069 (0.447)	−0.069	−0.79 (0.576)	0.790	0.988 (0.484)	**0.012**
3Trees-CTree	3.393 (0.862)	1.607	1.978 (0.084)	**0.022**	0.005 (0.064)	**−0.005**	2.817 (0.119)	0.183	0.996 (0.068)	0.004	1.384 (0.479)	0.616	−0.934 (0.424)	0.934	0.650 (0.665)	0.350
3Trees-EvTree	5.375 (0.635)	**−0.375**	2.206 (0.081)	−0.206	0.009 (0.045)	−0.009	3.003 (0.045)	**−0.003**	1.003 (0.048)	−0.003	2.059 (0.363)	**−0.059**	−0.134 (0.456)	**0.134**	0.950 (0.407)	0.050
LMMs	6.005 (0.582)	−1.005	3.585 (0.084)	−1.585	0.022 (0.084)	−0.022	2.989 (0.084)	0.011	1.007 (0.090)	−0.007	2.061 (0.436)	−0.061	−1.635 (0.436)	1.635	0.943 (0.518)	0.057
3Trees-TSCTree	6.021 (0.356)	−1.021	3.585 (0.084)	−1.585	0.022 (0.084)	−0.022	2.989 (0.084)	0.011	1.007 (0.090)	−0.007	2.062 (0.419)	−0.062	−1.639 (0.421)	1.639	0.949 (0.500)	0.051
**Scenario M3**																
True Value	5.000	–	2.000	–	0.000	–	2.000	–	2.000	–	3.000	–	0.000	–	2.000	–
3Trees-CART (cp=0.0001)	2.832 (1.329)	2.168	2.032 (0.142)	−0.032	−0.001 (0.071)	**0.001**	1.997 (0.070)	**0.003**	2.011 (0.076)	**−0.011**	3.058 (0.446)	−0.058	−0.582 (0.442)	0.582	2.058 (0.504)	−0.058
3Trees-CART (cp=0)	2.668 (1.425)	2.332	2.031 (0.142)	−0.031	−0.004 (0.071)	0.004	1.996 (0.070)	0.004	2.011 (0.076)	**−0.011**	3.065 (0.450)	−0.065	−0.572 (0.441)	**0.572**	2.062 (0.507)	−0.062
3Trees-CTree	3.782 (1.288)	**1.218**	−0.222 (0.109)	2.222	0.008 (0.094)	−0.008	1.301 (0.124)	0.699	1.897 (0.154)	0.103	1.568 (0.594)	1.432	−0.626 (0.343)	0.626	0.249 (1.105)	1.751
3Trees-EvTree	20.035 (0.864)	−15.035	2.007 (0.112)	**−0.007**	0.006 (0.087)	−0.006	1.996 (0.064)	0.004	2.012 (0.068)	−0.012	3.031 (0.375)	**−0.031**	−0.667 (0.418)	0.667	2.041 (0.417)	**−0.041**
LMMs	6.787 (0.520)	−1.787	2.015 (0.144)	−0.015	0.006 (0.145)	−0.006	2.005 (0.144)	−0.005	2.031 (0.155)	−0.031	3.037 (0.388)	−0.037	−0.727 (0.39)	0.727	2.065 (0.458)	−0.065
3Trees-TSCTree	6.908 (0.321)	−1.908	2.014 (0.144)	−0.014	0.006 (0.144)	−0.006	2.005 (0.144)	−0.005	2.031 (0.155)	−0.031	3.042 (0.374)	−0.042	−0.732 (0.376)	0.732	2.066 (0.440)	−0.066

**Fig. 3:**
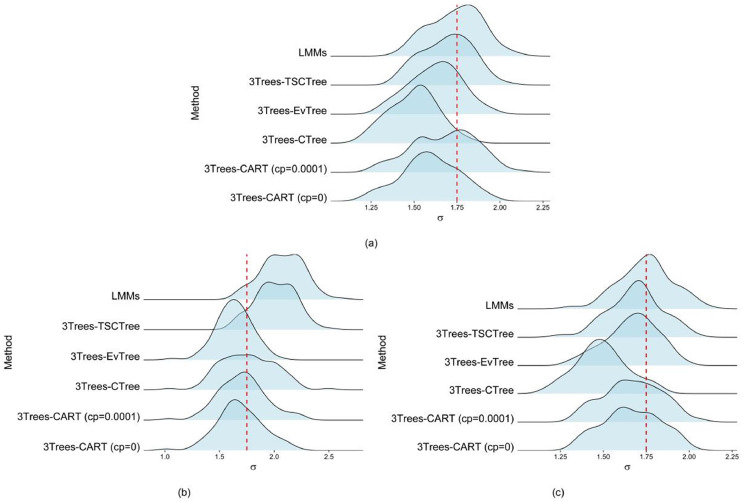
Density ridgeline plots of standard deviations corresponding to random effects (a) scenario M1, (b) scenario M2 and (c) scenario M3.

**Fig. 4:**
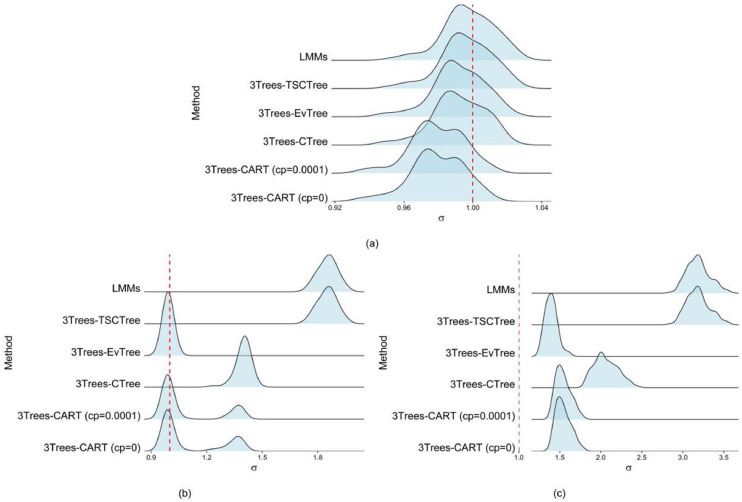
Density ridgeline plots of standard deviations corresponding to random error (a) scenario M1, (b) scenario M2 and (c) scenario M3.

In addition to estimating fixed effects, we also focus on comparing the variance within and between groups of main interest, as accurately estimating variance components is critically important [[32], [33], [34]]. It is due to when variance components are estimated precisely, it enhances the model's ability to capture the true underlying data structure. Furthermore, this procedure evaluates the performance of the REML method to estimate variance components. Fig. 3, Fig. 4 (ridgeline plots) display the distributions of the standard deviations of the random effects and random errors for all corresponding methods used, respectively. The true values of these standard deviations are represented by the red vertical dashed lines in each figure, $\sigma_{u}=1.75$ and $\sigma_{\varepsilon }=1,$ respectively. For scenario M1 (Fig. 3a), overall, the LMMs and 3Trees-TSCTree appear to provide the most accurate and consistent standard deviations of level-2 variance estimations among the methods evaluated, closely followed by 3Trees-EvTree and 3Trees-CTree. The 3Trees-CART methods show greater variability, especially with a complexity parameter of 0.0001.

Regarding scenario M2 (Fig. 3b), the 3Trees-CTree method exhibits the best performance in estimating the primary standard deviations of variance of interest, showing minimal bias. Meanwhile, the 3Trees-EvTree method provides greater consistency in the variability estimates considered. However, both the 3Trees-TSCTree and LMMs methods display the largest biases, indicating a tendency to overestimate the standard deviations of variance. For nonlinear functional forms (scenario M3-Fig. 3c), the 3Trees-EvTree method provides the most consistent and least biased results. The 3Trees-CTree method is also reasonably reliable; however, it appears to underestimate the standard deviations. In contrast, the 3Trees-TSCTree method shows the least bias. The 3Trees-CART implies slightly more variability in the estimates.

Besides discussing the random effect components, the variability of the estimated random error components is analyzed. The findings regarding the standard error of the residual variance, derived from 100 runs, are illustrated in Fig. 4. The true value for the standard error of the random error variance is represented by a vertical red dashed line at 1 for each ridgeline plot. Almost no significant differences exist among the models in estimating error variance under linear conditions (scenario M1), except for the CART model, which is slightly more biased than the other models. The EvTree method shows remarkable effectiveness in estimating variance under quasi-linear function conditions (scenario M2). It possesses minimal bias and superior reliability relative to other methods. In contrast, the LMMs and TSCTree methods display significant bias and often overestimate variance, a trend also observed in the CTree method. Furthermore, the presence of multiple peaks indicates that the CART method may lack reliability and stability in variance estimation. In contrast to the M1 and M2 settings, all methods are inclined to overestimate residual variance under nonlinear function conditions.

**Fig. 5:**
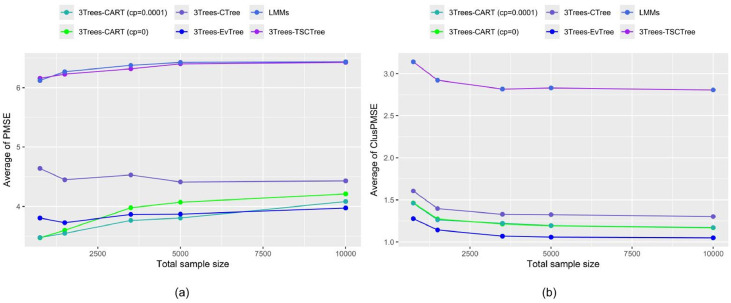
The average of MSE of predictions of testing data with and without random effects under scenario M2 (100 repetitions) (a) PMSE (b) ClusPMSE.

The results from the sample size variation are displayed in order to assess the consistent behavior of predictions of 3Trees model (CART, EvTree, CTree and TSCTree) and LMMs methods as the number of observations increase. In Fig. 5, the average PMSE (the left-hand side figure) and ClusPMSE (the right-hand side figure) values for the semilinear functional forms (M2 scenarios) with unequal sample sizes $\left(n_{tot}=750,\;1500,\;3500,\;5000,\;10000\right)$are presented. $n_{tot}=750\;\left(J=50,\;n_{j}=15\right),\;1500\;\left(J=50,\;n_{j}=30\right),\;3500\;\left(J=70,\;n_{j}=50\right),\;5000\left(J=100,\;n_{j}=50\right),\;$and $10000\;(J=200,\;n_{j}=50)$are set. For scenario M2, ρ=0.8 and ICC=0.75 are utilized. We further note that as the sample size enlarges, all regressors consistently perform better and better in terms of clusPMSE (except for PMSE criteria, which are less stable), and the value of this criteria is close to zero. Clearly, increasing the number of units has a favorable impact, reducing relative error prediction. Moreover, the proposed EvTree method is consistently superior to all the other ones in terms of clusPMSE (especially when a number of sample sizes is larger, starting at $n_{\text{tot}}=750$ and decreasing monotonically). However, when criteria excluding random effects for prediction are applied, the EvTree method outperforms only when the sample size sets 10,000 $\left(J=200,\;n_{j}=50\right)$observations. The performance of 3Trees-CART (cp=0) is similar to that of 3Trees-CART (cp=0.0001) (the lines overlap). Both methods rank second in their consistency in reducing prediction error. Then, the 3Trees-CTree method produces less accurate outcomes relative to the CART algorithm. On the other hand, LMMs and 3Trees-TSCTree (the lines coincide) perform worse than the other methods.

**Fig. 6:**
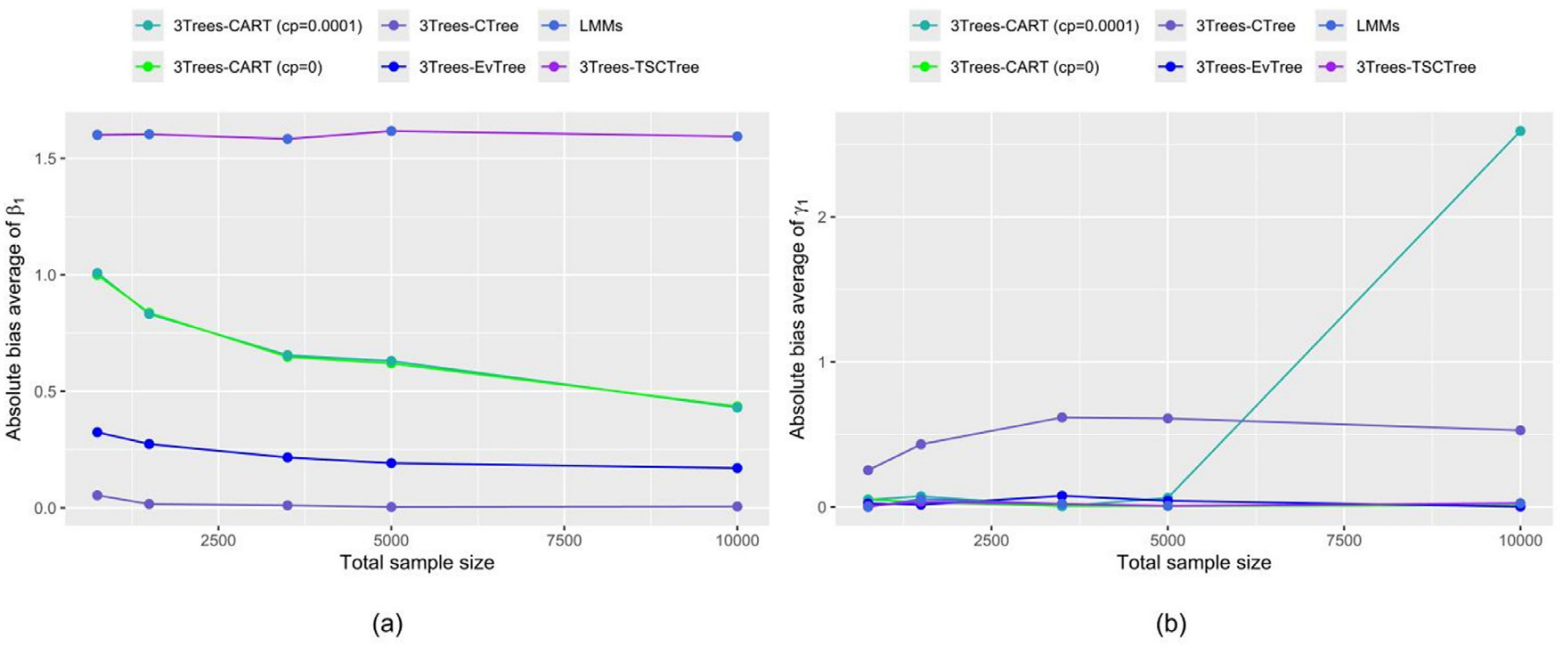
Absolute bias average of fixed effects of estimated parameters with various sample sizes (a) $\beta_{1}$ (b)$\;\gamma_{1}$ .

In addition to investigate the behavior of the accuracy in prediction, the behavior of estimated fixed-effect parameters across varying numbers of observations are considered. Fig. 6 shows the absolute bias averages of the estimates of level-1 and level-2 parameters for unequal sample sizes during scenario M2 when the ρ and ICC set at 0.8 and 0.75, respectively. Only a single fixed-effect parameter is shown at level 1 and level 2, denoted as $\beta_{1}$ and $\gamma_{1}$ for each. As a part of the 3Trees-EvTree and 3Trees-CTree analysis, as expected, the increase in sample size results a decrease in the bias absolute for considered estimators, although the 3Trees-CTree method shows a slight inconsistency in estimating level-2 parameters. This confirms that as the sample size increases, the estimated parameters correspond more closely to their true values in the simulation design, emphasizing the large sample sizes in achieving precise parameter estimation. The LMMs and 3Trees-TSCTrees methods portray similar performance, while the 3Trees-CART (cp = 0.0001) appears unstable when $n_{tot}=10000$.

**Fig. 7:**
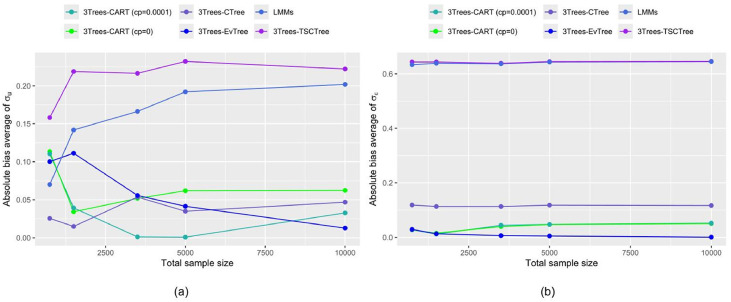
Absolute bias average of random effects of estimated parameters with various sample sizes (a) $\sigma_{u}$ (b) $\sigma_{\varepsilon }$.

Information on the estimated cluster and residual variance components are provided when sample sizes are moderate to large. Fig. 7 shows the results of the simulation study for variance estimation (within and between) under each of the above scenarios based on simulated unequal sample size from the assumed M2 model. The absolute bias average of variance parameters for each sample size considered is calculated. The left is random effects and the right one is random errors. Simulation results show that the accuracy of estimating the $\sigma_{u}$ and $\sigma_{\varepsilon }$ is increased when the sample size is larger. As n increases, the performance of 3Trees-Evtree methods for estimating $\sigma_{u}$and $\sigma_{\varepsilon }$is improved (absolute biases decrease as n increases). This confirms that 3Trees-EvTree produces good estimates of random effects and their standard errors. Meanwhile, LMMs, 3Trees-TSCTree, 3Trees-CART (cp=0), and 3Trees-CTree are often less stable about random effect estimation, except for the 3Trees-CART (cp=0.0001) method, which offers slightly greater consistency.

Finally, to explore the breakdown point of the proposed methods, an extra simulation scenario is designed. This simulation includes a random slope only for the explanatory variable $X_{1}$ in the scenario models. The results of the performance of error prediction for all methods are shown in Table 8, Table 9 (Appendix A). Also, the distribution of bias estimation (Table 11, Table 12-Appendix B) and the error prediction (Fig. 10-Appendix C) are presented. We observe that the performance of our proposed models underlying random slope models is almost the same as the random intercept models.

### Case study: Expenditure per Capita

The empirical dataset, household expenditure per capita dataset of West Java, were drawn from the National Social Economic Survey (SUSENAS) 2021, Central Agency of Statistics (BPS), Indonesia. The dataset involves data from 25,813 households, which represents the total following the removal of incomplete records. The dependent variable is household expenditure per capita. Meanwhile, a hierarchically structured dataset, where households were nested within a village. This situation make it possible to collect variables on both the level of the households (first level) and the level of villages (second level).

Therefore, our model includes ten explanatory variables for the individual level. The study area consists of 2200 villages in West Java Province, distributed among 29 regencies and cities. These villages are designated as clusters for the random effects in our model. The predictor variables for the cluster level were sourced from the Village Potential Data Collection (PODES), BPS, 2021. Moreover, there are five variables for village level (Table 5).

**Table 5:** Explanatory variables.

Variables	Label	Type of variable
*Household level*		
Gender	gender	factor (1=male, 2=female)
Age	age	continues
Level of education	education	factor(1: not graduated, 2: primary school, 3: junior high school, 4: senior high school, 5: Diploma, 6: Bachelor's degree and graduate studies)
Primary employment status	employment_status	factor (1: Self-employed, 2: Self-employed with temporary or unpaid workers, 3: Self-employed with regular or paid employees, 4: Worker/Employee/Staff, 5: Freelancer, 6: Unpaid family worker, 7:Unemployed)
Household size	member	continues
Regional status	regional_status	factor (1: city, 2:village)
Social security BPJS status	social_security_status	factor (1: yes, 2: no)
Housing tenure status	housing_tenure	factor (1: Personal ownership, 2: contract/rent, 3:Free of charge, 4:official residence)
Lightning sources	lightning_source	factor (1:PLN electricity with a meter, 2:PLN electricity without a meter, 3:Non-PLN power source,4: Non-electricity)
Regular government assistence status	regular_assistence	factor (1: yes, 2: no)
*Village level*		
Household income sources	income_source	factor (1: Agriculture and Natural Resources, 2: Energy and Utilities, 3:Manufacturing and Construction, 4: Trade and Repair, 5: Transportation and Accommodation, 6:Financial Services and Real Estate, 7: Government, Education, and Health, 8: Other Services)
Type of transportation Infrastructure to/from Agricultural Production Centers	road_type	factor (1: Asphalt/Concrete, 2:Gravelled (gravel, stone, etc.), 3:Land, 4: Water, 5: others)
Drinking Water Sources (group)	water_source_gr	factor (1: purified drinking water, 2: Water from the Supply and Distribution System, 3: Water from an Unprotected and Natural Source)
Number of educational facilities	num_education	continues
Number of health facilities	num_health	continues

The 3Trees-CART with cp=0, 3Trees-EVTree, 3Trees-CTree, and LMMs method for the random intercept assumption were applied to examine the determinants of household expenditure per capita. However, TSCTree and CART with cp=0.0001 methods were omitted for fitting and predicting data. The tuning parameters for each method according to the simulation settings are configured. The results of prediction performances are presented in Table 10. As one can see, we also used four different criteria (MSE, clusMSE, PMSE, clusPMSE) to compare the prediction of household expenditure per capita (Table 6). According to Table 6, the 3Trees-EvTree, which had the lowest error prediction value in terms of clusMSE and clusPMSE, was a slightly better fit and predicted with the data compared with the other methods.

**Table 6:** Prediction accuracy.

Model	MSE	clusMSE	PMSE	clusPMSE
3Trees-EvTree	1.489	1.224	1.658	1.533
3Trees-CART	1.482	1.226	1.654	1.534
3Trees-CTree	1.505	1.247	1.687	1.566
LMMs	1.566	1.282	1.750	1.611

The estimated regression coefficients (linear and tree components) associated with the household and village level characteristics, standard error, and p-values are given in Table 7. Accordingly, results from the 3Trees-EvTree model revealed that age, education (junior high school, senior high school and over diploma), employment_status (self-employed with regular or paid employees, freelancer, and unemployed), social_security_status (no), member, housing_tenure (contract/rent and Free of charge), lightning_source (PLN electricity without a meter), regular_assistence (no), num_education, income_source (financial services and real estate; government, education, and health), water_source (water from the supply and distribution system; water from an unprotected and natural Source) were the significant predictors of household expenditure per capita in West Java (p-values of <5 %) . At the first level, for instance, households with higher education levels (diploma to doctorate) show a noticeably larger coefficient. This suggests that these households tend to have much higher per capita expenditures compared to those with no formal education.

**Table 7:** Parameter estimates for 3Trees-EvTree model of household-and village-level factors for expenditure per capita.

Variable	Parameter	Standard Error	pvalue
*Household level*			
Intercept	2.263	0.156	0.001*
Regional_status (village)	0.059	0.036	0.111
Gender (female)	0.053	0.035	0122
Age	0.002	0.001	0.038*
Education (primary school)	0.091	0.071	0.201
Education (junior high school)	0.224	0.076	0.003*
Education (senior high school)	1.024	0.109	0.001*
Education (diploma, undergraduate, graduate)	2.307	0.113	0.001*
Employment_status (self-employed with temporary or unpaid workers)	−0.046	0.041	0.269
Employment_status (self-employed with regular or paid employees)	0.609	0.059	0.001*
Employment_status (worker/employee/staff)	0.027	0.029	0.352
Employment_status (freelancer)	−0.127	0.038	0.001*
Employment_status (unpaid family worker)	−0.098	0.116	0.394
Employment_status (unemployed)	−0.143	0.039	0.001*
Social_security_status (no)	0.218	0.023	0.001*
Member	−0.187	0.011	0.001*
Housing_tenure (contract/rent)	−0.480	0.042	0.001*
Housing_tenure (free of charge)	−0.328	0.036	0.001*
Housing_tenure (official residence)	0.208	0.228	0.361
Lighting_source (PLN electricity without a meter)	−0.086	0.038	0.024*
Lighting_source (Non-PLN power source)	−0.158	0.212	0.456
Lighting_source (Non-electricity)	−0.451	0.360	0.211
regular_assistence (no)	0.155	0.047	0.001*
*Village Level*			
Num_education	0.019	0.004	0.001*
Num_health	0.004	0.003	0.211
Road_type (gravelled)	−0.036	0.043	0.400
Road_type (land)	−0.002	0.056	0.968
Road_type (water)	0.163	0.242	0.503
Road_type (others)	−0.078	0.185	0.674
Income_source (energy and utilities)	−0.237	0.449	0.598
Income_source (manufacturing and construction)	0.143	0.244	0.556
Income_source (trade and repair)	0.286	0.245	0.241
Income_source (transportation and accommodation)	0.411	0.277	0.138
Income_source (financial services and real estate)	0.571	0.274	0.037*
Income_source (government, education, and health)	1.004	0.276	0.001*
Income_source (other services)	0.327	0.249	0.189
Water_source (water from the supply and distribution system)	−0.083	0.029	0.005*
Water_source (water from an unprotected and natural source)	−0.113	0.047	0.017*
*Tree component*			
First tree, region R1.5	−0.988	0.112	0.001*
First tree, region R1.7	−0.952	0.055	0.001*
First tree, region R1.8	−0.646	0.061	0.001*
First tree, region R1.11	−0.239	0.064	0.002*
First tree, region R1.12	−0.549	0.078	0.001*
Second tree, region R2.3	−0.103	0.035	0.003*
Third tree, region R3.4	−0.683	0.099	0.001*
Third tree, region R3.5	−0.919	0.089	0.001*
*Random component*			
$\sigma_{u}$	0.408		
$\sigma_{\varepsilon }$	1.147		

Note: *Statistically significant at the 5 % confidence level.

**Fig. 8:**
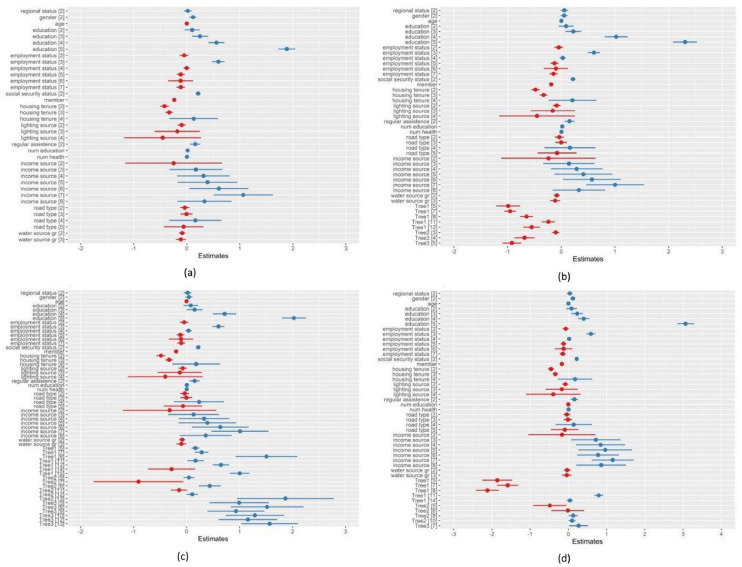
95 % confidence intervals of parameter estimations in the analysis of household expenditure percapita data using various methods; (a) LMMs, (b) 3Trees-EvTree, (c) 3Trees-CART, (d) 3Trees-CTree.

The standard deviation of random effect estimates revealed that there was more variation within the villages (1.147) than between the villages (0.408). Furthermore, the variation among rural areas was relatively more significant (ICC=0.112) on the level of expenditure per capita (see Table 7). In addition, confidence intervals for each parameter estimate are constructed. Coefficient plots in Fig. 8 visualize the interval estimation of coefficient regression under all models. The blue and red dot and the horizontal line (blue and red) mark the estimate and the 95 % confidence interval of the corresponding linear components and tree parts. These interval values provide an overview of the condition of the household expenditure per capita population. The confidence intervals of linear parameters for each model are not significantly different for p-value<0.05. However, for tree parts, the 3Trees-CART produces a wider confidence interval, while our proposed models are a little bit narrower than those of the 3Trees-CART.

**Fig. 9:**
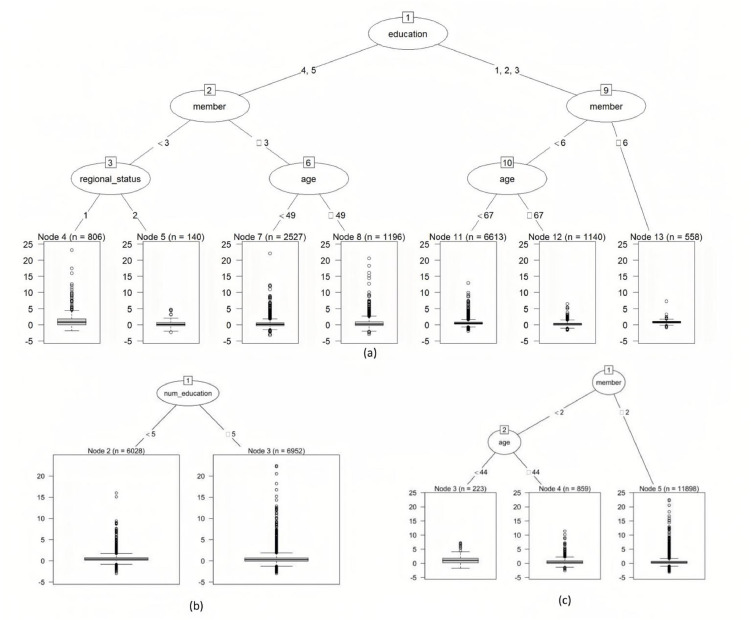
Tree generated by the proposed method (EvTree) for household expenditure per capita; (a)Tree 1, (b) Tree 2, and (c) Tree 3.

**Fig. 10:**
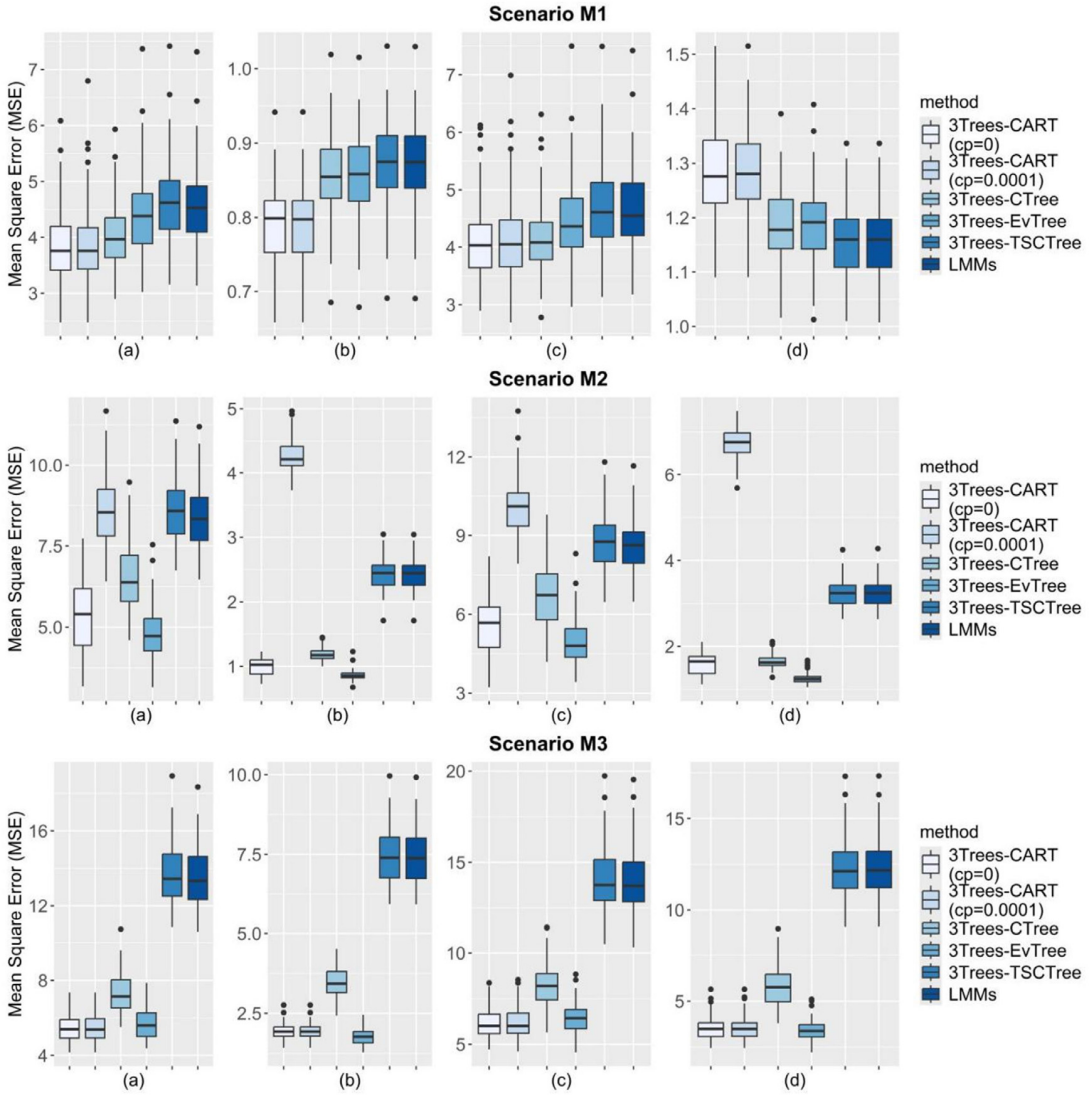
Boxplots for distributions of evaluation metrics under random slope models (a) MSE (b) ClusMSE (c) PMSE (d) ClusPMSE under random slope model.

To identify interaction terms among explanatory variables, tree structures ($T_{1},\;T_{2}$ and $T_{3}$) of the 3Trees-EvTree method for household expenditure per capita are depicted. These trees are shown in Fig. 9. For $T_{1}$ (see Fig. 9a), the selected split variable is level of education, household size (member), age and regional status. In both levels of education groups, the next split was based on household size (member). At the level of education with senior high school and higher education, a third split is regional status (node 4 and node 5) and age (node 7 and node 8). For the split at node 8, age<49 plays a significant role in influencing household expenditure per capita compared to age ≥ 49. Meanwhile, another part is the pathway when the level of education is primary and lower secondary education, including two paths from node 1 to node 11 and from node 1 to node 13. Note that these pathways show negatively impact to expenditure per capita of households. Moreover, for those who reported having fewer than 6 members and those with 6 members, even if the age score was below 67 or above 67, their expenditure per capita still maintained a low impact compared to households with senior high school or higher education levels.

Meanwhile, the 3Trees-CART model tends to generate a greater number of tree parameters or M-level factors $\left(M_{1}=8,\;M_{2}=7,\;M_{3}=7\right)$ compared to the 3Trees-EvTree $(M_{1}=7,\;M_{2}=2,\;M_{3}=3).$ This result is consistent with the fundamental characteristics of the EvTree algorithm to keep the depth of the tree small whenever the functional form of the dependence is assumed to be quasi-linear.

Then, the 3Trees-EvTree selected the number of educational facilities at $T_{2}$ as the only partitioning variable. Consequently, a single treatment interaction is depicted at the terminal nodes in Fig. 9(b). This tree highlights that the number of educational facilities with <5 and those with >5 have an almost equivalent impact on per capita household expenditure. Surprisingly, the $T_{3}$ plot (Fig. 9c) does not provide interactions between levels of predictor variables. The pathways only display member and age as partitioning variables. To capture interactions across levels, a higher maxdepth value may be necessary. However, this would increase model complexity, potentially making the tree more difficult to interpret.

## Conclusions

In this paper, we proposed EvTree and CTree algorithms to modify the tree selection step in the 3Trees algorithm. Our goal was to achieve more optimal trees through this selection process and to reduce the prediction error in terms of MSE, clusMSE, PMSE and clusPMSE. With regard to predictive accuracy (clusMSE and clusPMSE) and parameter estimations, the performances of our proposed model were evaluated over a variety of scenarios, where 3Trees-EvTree consistently show promising performance from both perspectives. Meanwhile, the 3Trees-CTree method only performed well after 3Trees-EvTree when the correlation among covariates was small for semilinear model scenario. Through the real data analysis, it could also be seen that 3Tree-EvTree outperformed in comparison with CART and CTree approaches when random components in prediction were considered.

## Limitations

Although the 3Trees-EvTree offers some benefits, this method is considerably more time-consuming compared to the other models, particularly when the number of observations is increased. Note that the optimal objective in machine learning applications is to balance the time-consuming nature of training data with the prediction accuracy of the desired outcomes. *Evtree* package require approximately 40–50 min and a main memory of 400 MB for fitting large datasets (see Grubinger et al. [23] and Zhang et al. [35]). As a result, a key challenge moving forward is to reduce running time, which may involve enhancing the specifications of the computers utilized or developing new algorithms to achieve this. On the other hand Evtree does not support to parallel computing. In addition, the 3Trees algorithm is only suitable for gaussian data (normal distribution). Thus, there is significant potential for further works of this method to cover non-Gaussian data (such as binary outcomes, beta distributions, or count data).

## Ethics statements

Dataset from SUSENAS and PODES are not publicly available to preserve individuals’ privacy under The Central Bureau of Statistics (Indonesia). These dataset may request access by visiting https://silastik.bps.go.id/v3/index.php/site/login/

## CRediT author statement

**Asrirawan**: Conceptualization, methodology, data curation, funding acquisition, formal analysis, and writing—original draft preparation. **Khairil Anwar Notodiputro**: supervision, data and analysis validation, and review and editing. **Budi Susetyo**: Investigation, resources, supervision, and review and editing. **Sachnaz Desta Oktarina**: Software development, Project administration, supervision, and final manuscript approval.

## Supplementary material *and/or* additional information [OPTIONAL]

None.

## Declaration of competing interest

The authors declare that they have no known competing financial interests or personal relationships that could have appeared to influence the work reported in this paper.

## Figures and Tables

**Table 8:** Results of the 100 simulation runs in terms of the averages and standard deviations of MSE, ClusMSE, PMSE dan ClusPMSE for six models under random slope model (X1). The smallest are marked in bold.

ρ*=0.8, ICC=0.75*					ρ*=0.8, ICC=0.375*				
	MSE	ClusMSE	PMSE	ClusPMSE		MSE	ClusMSE	PMSE	ClusPMSE
**Scenario M1**					**Scenario M1**				
3Trees-CART (cp=0.0001)	3.873 (0.71)	**0.790 (0.050)**	4.138 (0.734)	1.283 (0.080)	3Trees-CART (cp=0.0001)	**7.130 (0.657)**	4.013 (0.239)	8.560 (0.799)	6.236 (0.368)
3Trees-CART (cp=0)	**3.846 (0.663)**	0.790 (0.051)	**4.100 (0.699)**	1.283 (0.081)	3Trees-CART (cp=0)	7.137 (0.641)	**4.009 (0.243)**	8.571 (0.802)	6.236 (0.382)
3Trees-CTree	4.020 (0.593)	0.857 (0.054)	4.152 (0.624)	1.186 (0.073)	3Trees-CTree	7.800 (0.686)	4.318 (0.248)	**8.545 (0.811)**	5.857 (0.336)
3Trees-EvTree	4.415 (0.707)	0.857 (0.054)	4.474 (0.697)	1.189 (0.071)	3Trees-EvTree	8.214 (0.723)	4.339 (0.252)	8.694 (0.82)	5.790 (0.308)
LMMs	4.577 (0.683)	0.873 (0.054)	4.672 (0.701)	**1.160 (0.068)**	LMMs	8.528 (0.719)	4.412 (0.251)	8.835 (0.859)	**5.649 (0.296)**
3Trees-TSCTree	4.666 (0.693)	0.873 (0.054)	4.682 (0.697)	1.161 (0.068)	3Trees-TSCTree	8.631 (0.734)	4.422 (0.253)	8.848 (0.859)	5.651 (0.297)
**Scenario M2**					**Scenario M2**				
3Trees-CART (cp=0.0001)	8.574 (1.002)	4.275 (0.27)	10.102 (1.113)	6.727 (0.391)	3Trees-CART (cp=0.0001)	**8.404 (1.055)**	**4.242 (0.312)**	**9.814 (1.072)**	6.681 (0.421)
3Trees-CART (cp=0)	5.372 (1.145)	0.995 (0.136)	5.654 (1.183)	1.592 (0.225)	3Trees-CART (cp=0)	8.426 (1.063)	4.247 (0.308)	9.844 (1.086)	6.690 (0.410)
3Trees-CTree	6.542 (1.114)	1.184 (0.094)	6.790 (1.214)	1.645 (0.150)	3Trees-CTree	10.331 (1.186)	4.654 (0.299)	10.89 (1.063)	6.349 (0.348)
3Trees-EvTree	**4.820 (0.764)**	**0.860 (0.073)**	**4.971 (0.839)**	**1.252 (0.106)**	3Trees-EvTree	10.223 (1.261)	4.623 (0.29)	10.609 (1.186)	**6.283 (0.342)**
LMMs	8.397 (1.002)	2.430 (0.232)	8.587 (1.004)	3.241 (0.313)	LMMs	12.287 (1.115)	6.052 (0.421)	12.452 (1.027)	7.742 (0.508)
3Trees-TSCTree	8.654 (1.041)	2.433 (0.232)	8.719 (1.042)	3.241 (0.313)	3Trees-TSCTree	12.531 (1.146)	6.066 (0.424)	12.542 (1.032)	7.742 (0.507)
**Scenario M3**					**Scenario M3**				
3Trees-CART (cp=0.0001)	5.435 (0.722)	1.937 (0.235)	6.179 (0.853)	3.528 (0.594)	3Trees-CART (cp=0.0001)	**8.769 (0.779)**	5.268 (0.442)	10.498 (0.943)	8.336 (0.755)
3Trees-CART (cp=0)	**5.416 (0.703)**	1.935 (0.237)	**6.165 (0.840)**	3.523 (0.589)	3Trees-CART (cp=0)	8.770 (0.784)	**5.266 (0.441)**	**10.487 (0.939)**	**8.334 (0.746)**
3Trees-CTree	7.282 (1.006)	3.464 (0.472)	8.259 (1.138)	5.777 (1.028)	3Trees-CTree	11.072 (0.927)	7.044 (0.622)	12.554 (1.309)	10.388 (1.275)
3Trees-EvTree	5.720 (0.839)	**1.762 (0.241)**	6.450 (0.814)	**3.420 (0.560)**	3Trees-EvTree	10.228 (0.976)	5.819 (0.504)	11.293 (1.038)	8.643 (0.791)
LMMs	13.521 (1.493)	7.427 (0.847)	14.006 (1.610)	12.373 (1.603)	LMMs	17.936 (1.606)	11.276 (0.966)	18.372 (1.827)	16.895 (1.827)
3Trees-TSCTree	13.695 (1.517)	7.45 (0.853)	14.039 (1.643)	12.357 (1.602)	3Trees-TSCTree	18.102 (1.617)	11.321 (0.974)	18.395 (1.836)	16.872 (1.835)

**Table 9:** Results of the 100 simulation runs in terms of the averages and standard deviations of MSE, ClusMSE, PMSE dan ClusPMSE for six models under random slope model (slope X1). The smallest are marked in bold (continue).

ρ*=0.2, ICC=0.75*					ρ*=0.2, ICC=0.375*				
	MSE	ClusMSE	PMSE	ClusPMSE		MSE	ClusMSE	PMSE	ClusPMSE
**Scenario M1**					**Scenario M1**				
3Trees-CART (cp=0.0001)	3.903 (0.631)	0.787 (0.036)	4.127 (0.592)	1.290 (0.079)	3Trees-CART (cp=0.0001)	7.283 (0.681)	4.062 (0.264)	8.673 (0.737)	6.301 (0.370)
3Trees-CART (cp=0)	**3.855 (0.590)**	**0.787 (0.036)**	**4.086 (0.577)**	1.290 (0.079)	3Trees-CART (cp=0)	**7.280 (0.680)**	**4.060 (0.264)**	8.674 (0.743)	6.301 (0.377)
3Trees-CTree	4.053 (0.587)	0.852 (0.039)	4.153 (0.576)	1.199 (0.067)	3Trees-CTree	7.771 (0.643)	4.383 (0.265)	**8.516 (0.666)**	5.903 (0.342)
3Trees-EvTree	4.447 (0.623)	0.856 (0.038)	4.483 (0.63)	1.194 (0.067)	3Trees-EvTree	8.304 (0.699)	4.418 (0.267)	8.708 (0.720)	5.819 (0.328)
LMMs	4.641 (0.670)	0.872 (0.039)	4.719 (0.659)	**1.172 (0.065)**	LMMs	8.626 (0.739)	4.492 (0.265)	8.818 (0.740)	5.683 (0.310)
3Trees-TSCTree	4.743 (0.685)	0.872 (0.039)	4.729 (0.665)	1.173 (0.065)	3Trees-TSCTree	8.724 (0.749)	4.503 (0.266)	8.832 (0.746)	**5.682 (0.309)**
**Scenario M2**					**Scenario M2**				
3Trees-CART (cp=0.0001)	5.924 (1.153)	1.050 (0.104)	6.133 (1.061)	1.695 (0.171)	3Trees-CART (cp=0.0001)	8.574 (1.002)	**4.275 (0.270)**	10.102 (1.113)	6.727 (0.391)
3Trees-CART (cp=0)	5.903 (1.129)	1.050 (0.105)	6.124 (1.050)	1.696 (0.169)	3Trees-CART (cp=0)	**8.558 (0.977**)	4.277 (0.269)	10.087 (1.125)	6.726 (0.388)
3Trees-CTree	5.096 (0.848)	1.098 (0.162)	5.320 (0.857)	1.551 (0.231)	3Trees-CTree	8.676 (0.998)	4.613 (0.298)	**9.533 (1.103)**	**6.232 (0.397)**
3Trees-EvTree	**4.780 (0.773)**	**0.850 (0.068)**	**4.902 (0.752)**	**1.258 (0.114)**	3Trees-EvTree	10.204 (1.181)	4.658 (0.277)	10.724 (1.211)	6.265 (0.348)
LMMs	8.455 (0.928)	2.459 (0.221)	8.589 (0.98)	3.285 (0.321)	LMMs	12.235 (1.137)	6.064 (0.366)	12.499 (1.089)	7.69 (0.507)
3Trees-TSCTree	8.738 (0.944)	2.462 (0.221)	8.708 (0.986)	3.285 (0.322)	3Trees-TSCTree	12.49 (1.174)	6.079 (0.368)	12.623 (1.086)	7.691 (0.506)
**Scenario M3**					**Scenario M3**				
3Trees-CART (cp=0.0001)	5.612 (0.698)	2.109 (0.331)	**6.384 (0.772)**	3.678 (0.533)	3Trees-CART (cp=0.0001)	8.927 (0.969)	5.338 (0.482)	**10.595 (0.913)**	**8.458 (0.641)**
3Trees-CART (cp=0)	**5.605 (0.701)**	2.109 (0.330)	6.388 (0.780)	3.684 (0.533)	3Trees-CART (cp=0)	**8.919 (0.969)**	**5.336 (0.483)**	10.605 (0.916)	8.468 (0.644)
3Trees-CTree	8.112 (0.999)	4.238 (0.612)	9.218 (1.148)	7.029 (1.176)	3Trees-CTree	11.868 (1.405)	7.711 (0.831)	13.44 (1.265)	11.467 (1.159)
3Trees-EvTree	5.846 (0.735)	**1.875 (0.272)**	6.616 (0.753)	**3.500 (0.481)**	3Trees-EvTree	10.268 (0.966)	5.809 (0.511)	11.313 (1.083)	8.617 (0.744)
LMMs	13.742 (1.445)	7.603 (0.858)	14.085 (1.331)	12.356 (1.528)	LMMs	17.731 (1.656)	11.079 (1.026)	18.194 (1.619)	16.739 (1.413)
3Trees-TSCTree	13.907 (1.464)	7.627 (0.862)	14.103 (1.377)	12.343 (1.523)	3Trees-TSCTree	17.897 (1.663)	11.123 (1.033)	18.233 (1.621)	16.716 (1.409)

**Table 10:** Parameter averages, standard deviations and bias of estimated fixed-effect components produced by 3Trees and LMM (ρ=0.2 and ICC=0.375).

Parameter																
	$\beta_{0}$	Bias	$\beta_{1}$	Bias	$\beta_{2}$	Bias	$\beta_{3}$	Bias	$\beta_{4}$	Bias	$\gamma_{1}$	Bias	$\gamma_{2}$	Bias	$\gamma_{3}$	Bias
**Scenario M1**																
3Trees-CART (cp=0.0001)	−0.233 (1.335)	5.233	2.007 (0.078)	−0.007	0.008 (0.081)	−0.008	2.994 (0.079)	0.006	1.009 (0.108)	−0.009	3.014 (0.308)	−0.014	0.032 (0.304)	−0.032	0.974 (0.41)	0.026
3Trees-CART (cp=0)	−0.394 (1.455)	5.394	2.002 (0.08)	**−0.002**	0.007 (0.082)	−0.007	2.991 (0.078)	0.009	1.011 (0.108)	−0.011	3.007 (0.311)	**−0.007**	0.015 (0.313)	−0.015	0.953 (0.415)	0.047
3Trees-CTree	0.310 (1.583)	4.690	1.858 (0.095)	0.142	0 (0.056)	**0.000**	2.72 (0.152)	0.280	1.006 (0.109)	**−0.006**	1.062 (0.620)	1.938	−0.012 (0.211)	0.012	0.544 (0.526)	0.456
3Trees-EvTree	4.938 (0.807)	0.062	2.003 (0.061)	−0.003	0.009 (0.065)	−0.009	3.001 (0.061)	**−0.001**	1.011 (0.108)	−0.011	3.025 (0.236)	−0.025	0.002 (0.232)	**−0.002**	0.992 (0.413)	**0.008**
LMMs	4.998 (0.505)	**0.002**	1.998 (0.056)	**0.002**	0 (0.057)	**0.000**	3.001 (0.056)	**−0.001**	1.010 (0.109)	−0.010	3.014 (0.226)	−0.014	0.022 (0.231)	−0.022	0.980 (0.444)	0.020
3Trees-TSCTree	5.030 (0.309)	−0.030	1.998 (0.056)	**0.002**	0 (0.057)	**0.000**	3.001 (0.056)	**−0.001**	1.010 (0.109)	−0.010	3.015 (0.217)	−0.015	0.022 (0.222)	−0.022	0.984 (0.427)	0.016
**Scenario M2**																
3Trees-CART (cp=0.0001)	−0.266 (1.242)	5.266	3.120 (0.260)	−1.120	0 (0.071)	**0.000**	3.006 (0.069)	−0.006	0.998 (0.116)	0.002	1.899 (0.292)	0.101	−1.024 (0.525)	1.024	1.029 (0.487)	−0.029
3Trees-CART (cp=0)	−0.670 (1.400)	5.670	3.117 (0.260)	−1.117	0.002 (0.071)	−0.002	3.006 (0.069)	−0.006	1.000 (0.115)	**0.000**	1.901 (0.292)	0.099	−1.020 (0.535)	1.020	1.003 (0.488)	**−0.003**
3Trees-CTree	3.031 (1.038)	1.969	1.992 (0.097)	**0.008**	0.003 (0.059)	−0.003	2.674 (0.149)	0.326	0.991 (0.113)	0.009	1.045 (0.406)	0.955	−0.092 (0.344)	**0.092**	0.697 (0.459)	0.303
3Trees-EvTree	5.113 (0.725)	**−0.113**	2.213 (0.122)	0.213	0.002 (0.056)	−0.002	3.004 (0.056)	**−0.004**	0.999 (0.109)	0.001	1.944 (0.236)	0.056	−0.202 (0.382)	0.202	0.975 (0.425)	0.025
LMMs	6.057 (0.610)	−1.057	3.585 (0.069)	−1.585	0.008 (0.069)	−0.008	3.005 (0.069)	−0.005	0.991 (0.133)	0.009	1.962 (0.274)	**0.038**	−1.668 (0.278)	1.668	0.991 (0.537)	0.009
3Trees-TSCTree	5.992 (0.372)	−0.992	3.585 (0.069)	−1.585	0.008 (0.069)	−0.008	3.005 (0.069)	−0.005	0.991 (0.133)	0.009	1.959 (0.264)	0.041	−1.668 (0.268)	1.668	1.005 (0.517)	−0.005
**Scenario M3**																
3Trees-CART (cp=0.0001)	2.699 (1.254)	2.301	2.061 (0.234)	−0.061	0.010 (0.065)	−0.010	1.994 (0.064)	0.006	1.987 (0.123)	0.013	2.996 (0.334)	0.004	−0.581 (0.305)	0.581	1.980 (0.498)	**0.020**
3Trees-CART (cp=0)	2.551 (1.382)	2.449	2.036 (0.236)	−0.036	0.008 (0.065)	−0.008	1.994 (0.064)	0.006	1.985 (0.124)	0.015	3.030 (0.332)	−0.030	−0.589 (0.307)	0.589	2.037 (0.518)	−0.037
3Trees-CTree	3.382 (1.490)	1.618	0.221 (0.099)	1.779	−0.002 (0.076)	**0.002**	1.653 (0.161)	0.347	1.875 (0.195)	0.125	1.414 (0.579)	1.586	−0.459 (0.221)	**0.459**	0.814 (0.752)	1.186
3Trees-EvTree	19.932 (1.102)	−14.930	2.045 (0.165)	−0.045	−0.004 (0.075)	0.004	1.996 (0.062)	**0.004**	1.993 (0.121)	**0.007**	3.003 (0.246)	**−0.003**	−0.640 (0.323)	0.640	1.968 (0.423)	0.032
LMMs	7.054 (0.526)	−2.054	2.011 (0.095)	**−0.011**	0.010 (0.095)	−0.010	1.992 (0.095)	0.008	1.977 (0.183)	0.023	3.014 (0.239)	−0.014	−0.646 (0.237)	0.646	1.955 (0.464)	0.045
3Trees-TSCTree	7.039 (0.329)	**−2.039**	2.011 (0.095)	**−0.011**	0.010 (0.095)	−0.010	1.992 (0.094)	0.008	1.977 (0.183)	0.023	3.014 (0.231)	−0.014	−0.645 (0.229)	0.645	1.958 (0.448)	0.042

**Table 11:** Parameter averages, standard deviations and bias of estimated fixed-effect components produced by 3Trees and LMMs (ρ=0.8 and ICC=0.75) (random slope model).

Parameter																
	$\beta_{0}$	Bias	$\beta_{1}$	Bias	$\beta_{2}$	Bias	$\beta_{3}$	Bias	$\beta_{4}$	Bias	$\gamma_{1}$	Bias	$\gamma_{2}$	Bias	$\gamma_{3}$	Bias
**Scenario M1**																
3Trees-CART (cp=0.0001)	1.634 (1.179)	3.366	1.999 (0.166)	**0.001**	−0.010 (0.092)	0.010	3.000 (0.089)	**0.000**	1.011 (0.076)	−0.011	2.885 (0.510)	0.115	0.157 (0.506)	−0.157	1.000 (0.536)	**0.000**
3Trees-CART (cp=0)	1.463 (1.254)	3.537	1.998 (0.166)	0.002	−0.010 (0.091)	0.010	3.007 (0.088)	−0.007	1.012 (0.076)	−0.012	2.917 (0.518)	0.083	0.117 (0.508)	−0.117	1.003 (0.529)	−0.003
3Trees-CTree	1.774 (1.385)	3.226	1.913 (0.171)	0.087	0.004 (0.073)	**−0.004**	2.879 (0.132)	0.121	1.002 (0.078)	**−0.002**	1.497 (0.669)	1.503	0.010 (0.397)	**−0.010**	0.812 (0.534)	0.188
3Trees-EvTree	5.055 (0.701)	−0.055	1.977 (0.159)	0.023	0.007 (0.078)	−0.007	3.012 (0.076)	−0.012	1.006 (0.077)	−0.006	2.919 (0.428)	0.081	0.077 (0.405)	−0.077	1.063 (0.461)	−0.063
LMMs	5.054 (0.565)	−0.054	1.983 (0.159)	0.017	0.005 (0.073)	−0.005	3.007 (0.073)	−0.007	1.004 (0.078)	−0.004	2.965 (0.431)	0.035	0.083 (0.431)	−0.083	1.063 (0.506)	−0.063
3Trees-TSCTree	4.994 (0.346)	**0.006**	1.983 (0.157)	0.017	0.005 (0.073)	−0.005	3.007 (0.073)	−0.007	1.004 (0.078)	−0.004	2.973 (0.409)	**0.027**	0.072 (0.408)	−0.072	1.054 (0.479)	−0.054
**Scenario M2**																
3Trees-CART (cp=0.0001)	0.704 (1.539)	4.296	3.006 (0.293)	−1.006	−0.007 (0.088)	0.007	3.002 (0.087)	−0.002	0.987 (0.090)	0.013	2.030 (0.598)	−0.030	−1.019 (0.658)	1.019	1.013 (0.630)	−0.013
3Trees-CART (cp=0)	0.588 (1.598)	4.412	2.997 (0.292)	−0.997	−0.005 (0.087)	0.005	3.001 (0.086)	**−0.001**	0.987 (0.090)	0.013	2.010 (0.591)	**−0.010**	−0.977 (0.670)	0.977	0.946 (0.638)	0.054
3Trees-CTree	3.491 (0.874)	1.509	2.013 (0.217)	**−0.013**	−0.002 (0.085)	**0.002**	2.806 (0.149)	0.194	0.981 (0.091)	0.019	1.530 (0.527)	0.470	−0.953 (0.489)	0.953	0.800 (0.740)	0.200
3Trees-EvTree	5.556 (0.886)	**−0.556**	2.347 (0.197)	−0.347	0.003 (0.073)	−0.003	2.994 (0.073)	0.006	0.994 (0.078)	**0.006**	1.900 (0.448)	0.100	−0.119 (0.556)	**0.119**	1.011 (0.500)	**−0.011**
LMMs	5.977 (0.698)	−0.977	3.672 (0.222)	−1.672	−0.006 (0.121)	0.006	3.003 (0.121)	−0.003	1.013 (0.130)	−0.013	1.903 (0.525)	0.097	−1.316 (0.521)	1.316	0.972 (0.620)	0.028
3Trees-TSCTree	5.910 (0.430)	−0.910	3.672 (0.220)	−1.672	−0.006 (0.121)	0.006	3.003 (0.121)	−0.003	1.013 (0.129)	−0.013	1.906 (0.499)	0.094	−1.318 (0.494)	1.318	0.986 (0.588)	0.014
**Scenario M3**																
3Trees-CART (cp=0.0001)	3.000 (1.292)	2.000	2.016 (0.274)	−0.016	0.032 (0.114)	−0.032	1.985 (0.111)	0.015	1.981 (0.118)	0.019	3.154 (0.545)	−0.154	−0.764 (0.53)	0.764	1.826 (0.631)	0.174
3Trees-CART (cp=0)	2.801 (1.361)	2.199	2.017 (0.273)	−0.017	0.029 (0.114)	−0.029	1.985 (0.111)	0.015	1.982 (0.118)	0.018	3.160 (0.549)	−0.160	−0.753 (0.533)	0.753	1.890 (0.632)	0.110
3Trees-CTree	4.131 (1.347)	**0.869**	0.061 (0.232)	1.939	−0.010 (0.144)	0.010	1.306 (0.195)	0.694	1.844 (0.241)	0.156	1.838 (0.673)	1.162	−0.633 (0.398)	**0.633**	1.092 (0.896)	0.908
3Trees-EvTree	16.707 (1.053)	−11.707	2.002 (0.236)	**−0.002**	0.002 (0.121)	**−0.002**	1.998 (0.103)	**0.002**	1.981 (0.111)	0.019	3.162 (0.427)	−0.162	−0.806 (0.487)	0.806	1.895 (0.474)	0.105
LMMs	6.868 (0.633)	−1.868	2.014 (0.309)	−0.014	0.015 (0.209)	−0.015	1.998 (0.208)	**0.002**	1.997 (0.224)	0.003	3.120 (0.467)	−0.120	−0.776 (0.47)	0.776	1.939 (0.553)	0.061
3Trees-TSCTree	6.828 (0.387)	−1.828	2.014 (0.306)	−0.014	0.016 (0.208)	−0.016	1.998 (0.208)	**0.002**	1.998 (0.223)	**0.002**	3.115 (0.445)	**−0.115**	−0.764 (0.447)	0.764	1.943 (0.525)	**0.057**

**Table 12:** Parameter averages, standard deviations and bias of estimated fixed-effect components produced by 3Trees and LMMs (ρ=0.2 and ICC=0.375) (random slope model).

Parameter																
	$\beta_{0}$	Bias	$\beta_{1}$	Bias	$\beta_{2}$	Bias	$\beta_{3}$	Bias	$\beta_{4}$	Bias	$\gamma_{1}$	Bias	$\gamma_{2}$	Bias	$\gamma_{3}$	Bias
**Scenario M1**																
3Trees-CART (cp=0.0001)	−1.124 (1.511)	6.124	1.979 (0.199)	0.021	−0.001 (0.129)	**0.001**	3.003 (0.136)	−0.003	0.992 (0.170)	0.008	2.934 (0.370)	0.066	0.017 (0.369)	−0.017	0.902 (0.505)	0.098
3Trees-CART (cp=0)	−1.054 (1.534)	6.054	1.981 (0.201)	0.019	0.003 (0.128)	−0.003	2.998 (0.135)	**0.002**	0.995 (0.170)	0.005	2.938 (0.373)	0.062	0.022 (0.372)	−0.022	0.892 (0.511)	0.108
3Trees-CTree	0.229 (1.587)	4.771	1.712 (0.206)	0.288	−0.011 (0.089)	0.011	2.492 (0.234)	0.508	0.983 (0.172)	0.017	1.239 (0.656)	1.761	0.011 (0.245)	−0.011	0.581 (0.592)	0.419
3Trees-EvTree	4.562 (0.915)	0.438	1.978 (0.175)	0.022	−0.004 (0.105)	0.004	2.989 (0.106)	0.011	0.999 (0.172)	**0.001**	2.971 (0.279)	0.029	−0.021 (0.275)	0.021	0.881 (0.482)	0.119
LMMs	5.064 (0.613)	−0.064	1.982 (0.170)	**0.018**	−0.013 (0.090)	0.013	2.995 (0.090)	0.005	0.998 (0.174)	0.002	2.975 (0.281)	0.025	−0.001 (0.275)	**0.001**	0.922 (0.533)	**0.078**
3Trees-TSCTree	5.008 (0.375)	**−0.008**	1.982 (0.168)	**0.018**	−0.013 (0.090)	0.013	2.995 (0.090)	0.005	0.998 (0.173)	0.002	2.984 (0.266)	**0.016**	−0.001 (0.260)	**0.001**	0.922 (0.507)	**0.078**
**Scenario M2**																
3Trees-CART (cp=0.0001)	−0.924 (1.51)	5.924	3.441 (0.407)	−1.441	0.011 (0.116)	−0.011	3.003 (0.124)	−0.003	1.004 (0.176)	**−0.004**	1.982 (0.363)	0.018	−0.965 (0.576)	0.965	0.899 (0.574)	0.101
3Trees-CART (cp=0)	−1.020 (1.538)	6.020	3.440 (0.408)	−1.440	0.003 (0.116)	**−0.003**	3.001 (0.125)	**−0.001**	1.007 (0.176)	−0.007	1.978 (0.362)	0.022	−0.987 (0.587)	0.987	0.936 (0.579)	**0.064**
3Trees-CTree	2.477 (1.199)	2.523	1.976 (0.226)	**0.024**	0.004 (0.092)	−0.004	2.559 (0.213)	0.441	0.990 (0.177)	0.010	1.095 (0.447)	0.905	−0.267 (0.391)	**0.267**	0.654 (0.599)	0.346
3Trees-EvTree	5.738 (0.924)	**−0.738**	2.598 (0.311)	−0.598	0.015 (0.093)	−0.015	3.009 (0.095)	−0.009	1.012 (0.178)	−0.012	2.007 (0.312)	**−0.007**	−0.985 (0.356)	0.985	0.926 (0.550)	0.074
LMMs	5.945 (0.727)	−0.945	3.671 (0.209)	−1.671	0.013 (0.105)	−0.013	3.012 (0.105)	−0.012	1.016 (0.202)	−0.016	1.963 (0.327)	0.037	−1.337 (0.328)	1.337	0.917 (0.635)	0.083
3Trees-TSCTree	5.979 (0.443)	−0.979	3.670 (0.206)	−1.670	0.012 (0.105)	−0.012	3.012 (0.105)	−0.012	1.015 (0.202)	−0.015	1.967 (0.311)	0.033	−1.341 (0.311)	1.341	0.910 (0.603)	0.090
**Scenario M3**																
3Trees-CART (cp=0.0001)	1.608 (1.380)	3.392	1.895 (0.379)	0.105	0.003 (0.111)	−0.003	2.001 (0.107)	**−0.001**	2.021 (0.194)	−0.021	2.988 (0.392)	0.012	−0.620 (0.400)	0.620	2.041 (0.565)	**−0.041**
3Trees-CART (cp=0)	1.605 (1.380)	3.395	1.899 (0.377)	0.101	0 (0.111)	**0.000**	2.003 (0.107)	−0.003	2.019 (0.194)	−0.019	2.994 (0.392)	0.006	−0.621 (0.401)	0.621	2.045 (0.566)	−0.045
3Trees-CTree	3.472 (1.609)	**1.528**	0.343 (0.226)	1.657	0.001 (0.116)	−0.001	1.519 (0.236)	0.481	1.770 (0.384)	0.230	1.428 (0.633)	1.572	−0.438 (0.269)	**0.438**	1.111 (1.009)	0.889
3Trees-EvTree	16.663 (1.284)	−11.663	1.945 (0.285)	0.055	0.016 (0.104)	−0.016	1.980 (0.106)	0.020	2.014 (0.197)	**−0.014**	3.002 (0.307)	**−0.002**	−0.624 (0.306)	0.624	2.108 (0.506)	−0.108
LMMs	6.718 (0.682)	−1.718	1.974 (0.271)	**0.026**	0.007 (0.140)	−0.007	1.986 (0.141)	0.014	2.029 (0.271)	−0.029	3.029 (0.308)	−0.029	−0.615 (0.309)	0.615	2.062 (0.591)	−0.062
3Trees-TSCTree	6.712 (0.420)	−1.712	1.974 (0.267)	**0.026**	0.007 (0.139)	−0.007	1.985 (0.141)	0.015	2.029 (0.270)	−0.029	3.031 (0.292)	−0.031	−0.608 (0.292)	0.608	2.069 (0.559)	−0.069

## Data Availability

The authors do not have permission to share data.
